# The physiological cost of diazotrophy for *Trichodesmium erythraeum* IMS101

**DOI:** 10.1371/journal.pone.0195638

**Published:** 2018-04-11

**Authors:** Tobias G. Boatman, Phillip A. Davey, Tracy Lawson, Richard J. Geider

**Affiliations:** School of Biological Sciences, University of Essex, Colchester, United Kingdom; Mount Allison University, CANADA

## Abstract

*Trichodesmium* plays a significant role in the oligotrophic oceans, fixing nitrogen in an area corresponding to half of the Earth’s surface, representing up to 50% of new production in some oligotrophic tropical and subtropical oceans. Whilst *Trichodesmium* blooms at the surface exhibit a strong dependence on diazotrophy, colonies at depth or at the surface after a mixing event could be utilising additional N-sources. We conducted experiments to establish how acclimation to varying N-sources affects the growth, elemental composition, light absorption coefficient, N_2_ fixation, PSII electron transport rate and the relationship between net and gross photosynthetic O_2_ exchange in *T*. *erythraeum* IMS101. To do this, cultures were acclimated to growth medium containing NH_4_^+^ and NO_3_^-^ (replete concentrations) or N_2_ only (diazotrophic control). The light dependencies of O_2_ evolution and O_2_ uptake were measured using membrane inlet mass spectrometry (MIMS), while PSII electron transport rates were measured from fluorescence light curves (FLCs). We found that at a saturating light intensity, *Trichodesmium* growth was ~ 10% and 13% lower when grown on N_2_ than with NH_4_^+^ and NO_3_^-^, respectively. Oxygen uptake increased linearly with net photosynthesis across all light intensities ranging from darkness to 1100 μmol photons m^-2^ s^-1^. The maximum rates and initial slopes of light response curves for C-specific gross and net photosynthesis and the slope of the relationship between gross and net photosynthesis increased significantly under non-diazotrophic conditions. We attribute these observations to a reduced expenditure of reductant and ATP for nitrogenase activity under non-diazotrophic conditions which allows NADPH and ATP to be re-directed to CO_2_ fixation and/or biosynthesis. The energy and reductant conserved through utilising additional N-sources could enhance *Trichodesmium’s* productivity and growth and have major implications for its role in ocean C and N cycles.

## Introduction

In marine ecosystems, phytoplankton primary production is often limited by the bioavailability of fixed N [1–3], where N-sources (e.g. NO_3_^-^, NO_2_^-^, NH_4_^+^, urea etc) are quickly depleted by fast growing phytoplankton [4]. A significant fraction (~ 25 Tg N yr^-1^) of N in the euphotic zone is lost via sedimentation to the deep ocean as particulate organic nitrogen (PON), making NO_3_^-^ concentrations higher at greater depth [5–7]. Whilst areas of upwelling transport NO_3_^-^ into the euphotic zone, there are vast regions of the oligotrophic open oceans that are dependent on the input of new N from N_2_-fixing cyanobacteria. Among the most important marine diazotrophs are *Trichodesmium* sp., which can form extensive surface blooms in the tropical and subtropical oceans [8–12].

Previous studies have highlighted *Trichodesmium’s* capacity to assimilate various forms of combined N-sources [13–17]. It is commonly assumed that *Trichodesmium* obtains most of its nitrogen quota from N_2_ fixation, however field-based measurements of N_2_ fixation show wide temporal and spatial variability [18]. The causes of this variability remain unclear, but environmental factors such as the availability of combined nitrogen may be a contributing factor.

### Diazotrophy

Diazotrophic cyanobacteria are able to meet their daily nitrogen quota by fixing dinitrogen (N_2_).

$$N_{2}+\;16ATP+{8H}^{+}+{8e}^{-}\to \;{2NH}_{3}+H_{2}+\;16ADP+16Pi$$ (1)

While N_2_ fixation is an extremely energy demanding process, *Trichodesmium* incurs additional costs related to the protection of nitrogenase from the irreversible inhibition of photosynthetically evolved O_2_ [9, 19, 20]. The separation of O_2_ evolution and N_2_ fixation is regulated over a diurnal cycle of N_2_ fixation and photosynthesis [21], involving daily synthesis and degradation of nitrogenase [22, 23] and alternation of photosynthetic activity states [24]. Temporal separation occurs over short timescales, where peak rates of photosynthesis (~ 10 am) and N_2_ (~ 12 pm) fixation vary over a diel period. Spatial separation occurs via diazocytes, which are reversibly specialised cells for nitrogen fixation [25, 26]. Diazocytes contain the necessary proteins to perform photosynthetic CO_2_ fixation and N_2_ fixation. However, it has been suggested that when fixing N_2_, cells increase cyclic electron transport around PSI to enhance ATP synthesis [21, 24], thus allowing the cells to meet the energetic demands of N_2_ fixation (Eq 1).

### Uptake of additional N-sources

Like other facultative diazotrophic cyanobacteria spp., *Trichodesmium* can exploit other forms of nitrogen including NH_4_^+^, NO_3_^-^, urea and amino acids [16, 27]. These N compounds are transported into the cell via permeases, metabolised to NH_4_^+^ and then incorporated into carbon skeletons through the glutamine synthetase (GS) and glutamine 2-oxoglutarate aminotransferase (GOGAT) pathways. This process is mediated by nitrate reductase (Eq 2) and nitrite reductase (Eq 3).

$${NO}_{3}^{-}+\;{2e}^{-}+{2H}^{+}\to {NO}_{2}^{-}+\;H_{2}O$$ (2)

$${NO}_{2}^{-}+\;{6e}^{-}+{8H}^{+}\to {NH}_{4}^{+}+\;{2H}_{2}O$$ (3)

For cyanobacteria, nitrate reductase is located in the cytosol and uses NADPH to catalyse the transfer of two electrons. The NO_2_^-^ formed by nitrate reductase is further reduced to NH_4_^+^ via the transfer of six electrons. Thus, the reduction of NO_3_^-^ to NH_4_^+^ can be expressed as;

$${NO}_{3}^{-}+\;{8e}^{-}+{10H}^{+}\to {NH}_{4}^{+}+{3H}_{2}O$$ (4)

Amino acids are synthesised from ammonia (NH_3_) via the GS-GOGAT pathway. The initial GS pathway requires ATP and glutamate as a substrate;

$$Glutamate\;+\;{NH}_{3}+\;ATP\to \;Glutamine\;+ADP+Pi$$ (5)

where glutamine is subsequently transformed to 2-oxoglutarate and reduced using NADPH, forming two moles of glutamate.

2-Oxoglutarate+Glutamine→2[Glutamate] (6)

Thus, for every mole of glutamate produced, one mole each of NH_3_, NADPH, ATP and 2-oxoglutarate are required. Additionally, ATP is required for the active transport of inorganic NH_4_^+^ or NO_3_^-^ into the cell [28]. Different N-sources require different amounts of energy and reductant and as such can be ordered into a hierarchy of energy requirements; where diazotrophy requires the highest investment of electrons and ATP, followed by NO_3_^-^, NO_2_^-^ and then NH_3_.

### Utilising additional N-sources

Global warming is increasing sea surface temperatures (SSTs) which is enhancing water stratification and decreasing vertical mixing [29], potentially increasing the area of N-limited oceans. Whilst detrimental to many phytoplankton, a reduced flux of NO_3_^-^ into the upper mixed layer will increase the competitive advantage of diazotrophs for other limiting nutrients (i.e. Fe or P). *Trichodesmium* colonies have been observed migrating to the nutricline [30, 31] to facilitate the luxury uptake of polyphosphates before returning to the surface. Whilst at these depths, cells are exposed to NO_3_^-^ concentrations greater than those at the surface. As such, *Trichodesmium* colonies may be assimilating and storing (i.e. cyanophycin granules) more combined N than the blooms frequently measured on the surface [32]. This could have major implications for growth rates, primary productivity and biogeochemical cycles [33].

Our approach comprises a systematic experiment where *T*. *erythraeum* IMS101 was grown over long durations, at three N-source treatments, with controlled and well-defined growth conditions, ensuring fully acclimated, balanced growth had been achieved. Our aims were to assess the response of *T*. *erythraeum* IMS101 growth, light dependency of gross and net O_2_ photosynthesis, PSII electron transport rates and elemental composition to different N-sources; investigating the physiological cost of performing diazotrophy.

## Materials and methods

*T*. *erythraeum* IMS101 was semi-continuously cultured to achieve fully acclimated balanced growth at three N-source treatments (N_2_, NH_4_^+^ and NO_3_^-^), at a targeted 380 μatm CO_2_ concentration, saturating light intensity (400 μmol photons m^-2^ s^-1^), 12:12 light:dark (L:D) cycle and optimal temperature (26 °C ± 0.2) (3 treatments in total) for ~ 2 months (~ 30 generations).

### Experimental setup

Cultures were acclimated to the CO_2_ and light intensity for ~ 4 months (~ 60 generations) under diazotrophic conditions before the addition of NH_4_^+^ or NO_3_^-^. Cultures were gradually enriched over a 2/3-week period by increasing the dilution ratio of YBCII media containing NH_4_^+^ or NO_3_^-^ (100 μM).

*T*. *erythraeum* IMS101 was grown using YBCII medium [34] at 1.5 L volumes in 2 L pyrex bottles that were acid-washed and autoclaved prior to culturing. Daily growth rates were quantified from changes in baseline fluorescence (*F*_*o*_) measured between 09:00 to 10:30 on dark-adapted cultures (20 minutes) using a FRRfII FastAct Fluorometer System (Chelsea Technologies Group Ltd, UK). Cultures were regarded as fully acclimated and in balanced growth when both the slope of the linear regression of ln *F*_*o*_ versus time and the ratio of live cell to acetone extracted (method detailed below) baseline fluorescence (*F*_*o*_) were constant following every dilution with fresh YBCII medium. Cultures were kept at the upper section of the exponential growth phase through periodic dilution with new growth media at 3–5 day intervals. Illumination was provided side-on by fluorescent tubes (Sylvania Luxline Plus FHQ49/T5/840). Cultures were constantly mixed using magnetic PTFE stirrer bars and aerated with a filtered (0.2μm pore) air mixture at a rate of ~ 200 mL s^-1^. The CO_2_ concentration was regulated (± 2 μatm) by mass flow controllers (Bronkhorst, Newmarket, UK). CO_2_-free air was supplied by an oil free compressor (Bambi Air, UK) via a soda lime gas-tight column which was mixed with a 10% CO_2_ in-air mixture from a gas cylinder (BOC Industrial Gases, UK). The CO_2_ concentration was continuously monitored and recorded by an infra-red gas analyser (Li-Cor Li-820, Nebraska USA), calibrated weekly by a standard gas (BOC Industrial Gases).

Throughout all culturing, the inorganic carbon chemistry (S1 File) and dissolved inorganic NH_4_^+^ and NO_3_^-^ concentrations (S2 File) were determined prior to diluting with fresh media. Samples for elemental composition, photosynthesis-light response curves, fluorescence light curves (FLC), *in vivo* light absorption and acetylene reduction assays were collected at the same time of day, approximately 4 and 6 hours into the photo-phase of the L:D cycle.

### Measuring O_2_ exchange by membrane inlet mass spectrometry (MIMS)

Light dependent rates of O_2_ production and consumption were measured with a membrane inlet mass spectrometer (MIMS), using an ^18^O_2_ technique modified from McKew *et al*. [35] (S3 File). MIMS measurements consisted of three biological replicates per treatment (S4 File). Chlorophyll *a* concentrations at the point of sampling ranged from 80 to 245 μg Chl*a* L^-1^.

Changes in ^16^O_2_ and ^18^O_2_ and thus O_2_ consumption (U_o_) and O_2_ evolution (E_o_) were calculated using the following equations [36];

U0=-(1+O162O182)∙ΔO182Δt (7)

E0=ΔO162Δt-(O162O182)∙ΔO182Δt (8)

where U_o_ is the rate of O_2_ consumption calculated from the decrease of ^18^O_2_ over time (i.e. Δ ^18^O_2_/Δt), which takes into account the relative concentration of ^18^O_2_ compared to ^16^O_2_ (i.e. 1 + ^16^O_2_/^18^O_2_) and E_o_ is the rate of gross O_2_ evolution calculated from the increase in ^16^O_2_ over time (Δ^16^O_2_/Δt), where the decline of ^18^O_2_ (i.e. Δ^18^O_2_/Δt) and ^18^O_2_ is corrected for relative to the concentration of ^16^O_2_. Chlorophyll *a*- and C-specific rates were obtained by dividing U_0_ and E_0_ by the concentration of Chl*a* and particulate organic carbon, respectively. Rates were multiplied by 1.073 to spectrally correct to the culturing LEDs (S1 Fig).

Photosynthesis-light (P-E) curves for gross (E_0_^Chl(C)^) and net photosynthesis (P_net_^Chl(C)^ = E_0_^Chl(C)^-U_0_^Chl(C)^) were fitted to the equations from Platt and Jassby [37];

$$E_{0}^{Chl(C)}\;=\;E_{0m}^{Chl(C)}\cdot \left[1-e(\frac{-\alpha_{g}^{Chl(C)}\;∙\;E}{E_{0m}^{Chl(C)}})\right]$$ (9)

$${P_{net}}_{\;}^{Chl(C)}\;=\;{P_{net}}_{m}^{Chl(C)}\;\cdot \left[1-e(\frac{-\alpha_{n}^{Chl(C)}\;∙\;E}{{P_{net}}_{m}^{Chl(C)}})\right]+\;R_{d}^{Chl(C)}$$ (10)

where E_0m_^Chl(C)^ and P_netm_^Chl(C)^ are the maximum gross and net O_2_ evolution rates; α_g_^Chl(C)^ and α_n_^Chl(C)^ are the initial light-limited slopes for gross and net photosynthesis; R_d_ is the dark respiration rate; and E is the light intensity (μmol photons m^-2^ s^-1^). Curve fitting was performed on each replicate separately to calculate mean (± S.E.) curve fit parameterisations (Sigmaplot 11.0).

The maximum quantum efficiency of gross (ɸ_mgross_) and net (ɸ_mnet_) O_2_ evolution was calculated as follows;

$${ɸ}_{m}\;=\;\frac{\alpha_{g(n)}^{C}}{a_{eff}^{C}}$$ (11)

where the C-specific initial slope for gross (α_g_^C^) or net (α_n_^C^) O_2_ evolution was divided by the C-specific, effective light absorption coefficient (a_eff_^C^).

### Measuring nitrogenase activity by acetylene reduction

Acetylene reduction rates were measured using gas chromatography (ATI Unicam 610 series). Gaseous samples were injected into the GC column head (60 °C), carried via N_2_ gas through a Porapak N column (100 °C) to a flame ionising detector (100 °C). Peak areas of acetylene and ethylene were quantified by an integrated chromatograph data acquisition unit (Shimadzu C-R8A Integrator) and were converted into concentrations via an acetylene and ethylene standard curve performed with standard gases (Scientific and Technical Gases Ltd., UK). Triplicate 6 mL samples of each biological replicate culture were placed into 12 mL exetainer, screw capped glass vials (Labco Ltd, UK). Exactly 1.2 mL of the headspace was removed and replaced with a 1.2 mL sample of acetylene (BOC Industrial Gases, UK) (headspace = 20% acetylene). The vials were gently inverted for 1 minute before 250 μL of headspace was injected into the GC column for an initial measurement of acetylene and ethylene concentrations (T_0_). Vials were incubated at 26 °C and 400 μmol photons m^-2^ s^-1^ in an aluminium temperature block and were gently inverted every 10 minutes to prevent trichomes from settling on the bottom or aggregating at the meniscus. After 1 hour, a second 250 μL gaseous headspace was injected into the GC column for the post-incubation measurement (T_1_). Temperature and pressure was measured during each set of measurements and accounted for in the calculations. The rate of ethylene production was calculated with the assumption that the concentrations of acetylene and ethylene within the media were always in equilibrium to those in the headspace;

$${\Delta C}_{2}H_{2}\;=\;\frac{{C_{2}H_{2}}_{\left(T_{1}\right)}-{\;C_{2}H_{2}}_{\left(T_{0}\right)}}{t\;∙{\;V}_{\left(I\right)}}$$ (12)

where (ΔC_2_H_2_) is the ethylene production rate (μmol C_2_H_4_ h^-1^), C_2_H_2(T0)_ and C_2_H_2 (T1)_ are the ethylene concentrations in the headspace at the start (T_0_) and end (T_1_) of the incubation, V_(I)_ is the volume of gaseous sample injected into the GC column (L^-1^) and t is the incubation time (min).

N_2_ fixation rates were calculated to a Chl*a* (μmol N_2_ (mg Chl*a*)^-1^ h^-1^) and total carbon (μmol N_2_ (mg C)^-1^ h^-1^) basis;

$$N_{2}\;fixation\;=\left(\frac{{\Delta C}_{2}H_{2}}{\left[Chl\;a(C)\right]}∙\;10^{3}\right)∙0.25$$ (13)

where ΔC_2_H_2_ (μmol h^-1^) is divided by the Chl*a* or total carbon concentration (mg) and multiplied by 0.25 under the assumption that reduction of four moles of acetylene is equivalent to reduction of one mole of dinitrogen.

### Fluorescence light curves (FLCs)

A 2 mL sample of each replicate culture was used to measure a fluorescence light curve (FLC) [38]. The FLCs were measured with a FRRfII FastAct Fluorometer System, using a white LED actinic light source (Chelsea Technologies Group Ltd, UK). Each FLC lasted 1 hour; comprising 12 light steps which ranged from 10 to 1600 μmol photon m^-2^ s^-1^, each lasting 5 minutes in duration. The FLCs provided measurements of the light absorption cross-section of PSII photochemistry (σ_PII_ʹ), the average time constant for the re-opening of a closed PSII reaction centre (τ_f_ʹ) and the operating efficiency of PSII photochemistry (*F*_*q*_*ʹ/F*_*m*_*ʹ*);

$$\frac{F_{q}ʹ}{F_{m}ʹ}\;=\left[\frac{F_{m}ʹ-Fʹ}{F_{m}ʹ}\right]$$ (14)

where *F*_*m*_ʹ is the maximum fluorescence in the light-adapted state and *F*ʹ is the steady-state fluorescence at any point.

Photosystem II (PSII) electron transport rates were normalised to a Chl*a* (mol e^-^ (g Chl*a*)^-1^ h^-1^) and total carbon (mol e^-^ (g C)^-1^ h^-1^) basis;

$${ETR}^{Chl(C)}\;=\;\frac{F_{q}ʹ}{F_{m}ʹ}∙E∙\left(a^{Chl(C)}\cdot {FAQ}_{PII}\right)∙3600∙SCF$$ (15)

where *F*_*q*_*ʹ/F*_*m*_*ʹ* is the operating efficiency of PSII photochemistry; E is the light intensity (mol photons m^-2^ s^-1^), a^Chl(C)^ is the Chl*a*-specific (C-specific) effective light absorption (m^2^ g^-1^ Chl*a* and m^2^ g^-1^ C, respectively), FAQ_PII_ is the fraction of absorbed photons directed to PSII, which was set to 0.5 [39], with the assumption that the quantum yield of electron transport of one trapped photon within a reaction centre is equal to 1 [40]; 3600 converts seconds to hours and SCF is a spectral correction factor of 1.194, which converts electron transport rates to the culturing LED spectrum (S1 Fig).

ETR curves were modelled using a P-E equation [37], performed on each individual replicate using a Marquardt–Levenberg least squares algorithm to generate the best fit (R^2^ > 0.993);

$$ETR\;=\;{ETR}_{m}ʹ∙\left[1-e(\frac{-\alpha_{ETR}\;∙\;E}{{ETR}_{m}ʹ})e(\frac{-\beta_{ETR}\;∙\;E}{{ETR}_{m}ʹ})\right]$$ (16)

where ETR_m_ʹ is the hypothetical Chl*a*(C)-specific maximum electron transport rate that would be achieved if there was no photoinhibition (mol e^-^ (g Chl*a*(C))^-1^ h^-1^); α_ETR_ is the initial slope of the Chl*a*(C)-specific ETR-light curve (mol e^-^ (g Chl*a*(C))^-1^ h^-1^ (μmol photons m^-2^ s^-1^)^-1^); β_ETR_ is the parameter that accounts for downregulation and/or photoinhibition at supra-optimal light intensities (mol e^-^ (g Chl*a*(C))^-1^ h^-1^ (μmol photons m^-2^ s^-1^)^-1^); and E is the light intensity (μmol photons m^-2^ s^-1^).

The realised maximum PSII electron transport rate in the presence of photoinhibition (ETR_m_), light intensity at which ETR is maximal (E_opt_), the light-saturation parameter (E_k_) and the light inhibition parameter (E_p_) were calculated from the fitted parameters as follows:

ETRm=ETRm'∙(αETRαETR+βETR)∙(βETRαETR+βETR)βETRαETR (17)

Eopt=ETRm'αETR∙ln(αETR+βETRβETR) (18)

$$E_{k}\;=\;\frac{{ETR}_{m}}{\alpha_{ETR}}$$ (19)

$$E_{p}\;=\;\frac{{ETR}_{m}}{\beta_{ETR}}$$ (20)

The ratio of PSII electron transport to gross O_2_ evolution (E_0_) under light-limitation (Φ_eα_) and light-saturation (Φ_em_) were calculated as follow;

$$\Phi_{e\alpha }\;=\;\frac{\alpha_{ETR}}{\alpha_{g}}$$ (21)

$$\Phi_{em}\;=\;\frac{{ETR}_{m}}{E_{0m}}$$ (22)

### Cellular elemental composition and light absorption

Samples for determining particulate organic carbon (POC), nitrogen (PN) and phosphorus (PP) (S5 File), chlorophyll *a* (S6 File) and *in vivo* light absorption (S7 File) were collected with each MIMS measurement, with each sample being a biological replicate.

### Modelling the *in vivo* light absorption from pigment absorption spectra

*In vivo* light absorption was reconstructed using the light absorption spectra of Chl*a* and photoprotective carotenoids (PPC) taken from Woźniak *et al*. [41] and the light absorption spectra of phycourobilin (PUB1, PUB2, PUBx, PUB4, PUB5a, PUBb, PUB5d, PUB5g and PUB5j), phycoerythrin (PE1, PE2a, PE2b and PE3b), alloplastocyanin (APC) and plastocyanin (PC1 and PC2) taken from Küpper *et al*. [42] (S2 Fig).

The Chl*a*-specific light absorption coefficient was modelled as the sum of the contribution of all pigments;

$$a_{mod}^{Chl}(\lambda )\;=\;\sum_{i}∙\beta_{i}∙a_{i}(\lambda )$$ (23)

where a^Chl^_mod_ is the modelled *in vivo* light absorption at a specific wavelength (λ = 400–700 nm); β^i^ is the contribution of each pigment to a^Chl^_mod_ and a^i^ is the pigment-specific spectral absorption coefficient of pigment i, in m^2^ (g pigment i)^-1^.

The modelled *in vivo* light absorption spectra (a^Chl^_mod_ (λ)) was optimised to the measured spectra between 400 and 700 nm using a reduced sum of squares method (Sigmaplot 11.0). If a zero value was returned for a β^i^ parameter, that pigment was removed from the model and the curve fit reapplied.

## Results

### Inorganic C-chemistry, growth rate and cell composition

Balanced growth of *T*. *erythraeum* IMS101 was 0.34 d^-1^ when grown on N_2_, increasing by 10% and 13% when grown in the presence of NH_4_^+^ and NO_3_^-^, respectively (Table 1). Particulate C:N, C:P and N:P ratios were all influenced by the presence of additional N-sources. When compared to the N_2_ treatment, C:N decreased by 36% and 43% for the NH_4_^+^ and NO_3_^-^ treatments, respectively. Ratios of C:P and N:P were comparable between NH_4_^+^ and NO_3_^-^ treatments, but were significantly lower (~ 60% and 35%, respectively) compared to the N_2_ treatment (Table 1). Ratios of Chl*a*:C were 80% and 67% higher for the NH_4_^+^ and NO_3_^-^ treatments than for the N_2_ treatment, while Chl*a*:N was not significantly different between treatments (Table 1). Carbon and Chl*a*-specific N_2_ fixation rates were highest for the N_2_ treatment, decreasing significantly by 84% and 80% (Chl*a*-specific) and 73% and 68% (C-specific) for the NH_4_^+^ and NO_3_^-^ treatments, respectively (Table 1).

**Table 1:** The median (± S.E.) balanced growth rates and mean elemental stoichiometry and N_2_ fixation rates for *T*. *erythraeum* IMS101 when acclimated to three N-source conditions (N_2_, NH_4_^+^ and NO_3_^-^), at a target CO_2_ concentration (380 μatm), saturating light intensity (400 μmol photons m^-2^ s^-1^) and optimal temperature (26 °C).

Variables	Units	N_2_	NH_4_^+^	NO_3_^-^
Growth rate	d^-1^	0.340 (0.038)^[A]^	0.375 (0.011)^[B]^	0.384 (0.005)^[B]^
Elemental Stoichiometry				
C:N	mol:mol	6.9 (0.7)	4.4 (0.9)	3.9 (0.7)
C:P	mol:mol	122.6 (7.0)^[B]^	47.9 (2.4)^[A]^	36.9 (2.9)^[A]^
N:P	mol:mol	18.1 (1.3)^[B]^	11.8 (2.2)	9.9 (1.0)^[A]^
Chl*a*:C	mg:mol	134 (8)^[A]^	239 (4)^[B]^	222 (2)^[B]^
Chl*a*:N	mg:mol	906 (43)	1041 (209)	855 (154)
N_2_ Fixation				
Chl*a*-specific	μmol N (mg Chl*a*)^-1^ h^-1^	14.75 (1.66)^[B]^	2.35 (0.49)^[A]^	2.84 (0.44)^[A]^
C-specific	μmol N (mg C)^-1^ h^-1^	0.16 (0.01)^[B]^	0.04 (0.01)^[A]^	0.05 (0.01)^[A]^

Abbreviations; C:N, C:P and N:P ratios are mol:mol, Chl*a*:C and Chl*a*:N ratios are mg:mol (*n* = 3). Letters in parenthesis indicate significant differences between N-source treatments (One Way ANOVA, Tukey post hoc test; P < .05); where [B] is significantly greater than [A].

The inorganic carbon concentration, pH and alkalinity (A_T_) did not vary significantly amongst N-source treatments. Overall, CO_2_ drawdown ranged between 78 to 92 μatm from the target concentration (i.e. 380 μatm) for all N-source treatments (Table 2) and exhibited little variability over a diurnal cycle (S3 Fig). Inorganic N concentrations were > 1 μM for the N_2_ treatment and were ~ 8 μM for the NH_4_^+^ and NO_3_^-^ treatments at the point of dilution (Table 2).

**Table 2:** The growth conditions (± S.E.) for *T*. *erythraeum* IMS101 when cultured under three N-source conditions (N_2_, NH_4_^+^ and NO_3_^-^), at a target CO_2_ concentration (380 μatm), saturating light intensity (400 μmol photons m^-2^ s^-1^) and optimal temperature (26 °C).

Variables	Units	N_2_	NH_4_^+^	NO_3_^-^
pH	Total	8.18	8.18	8.19
H^+^	nM	6.6 (0.1)	6.6 (0.1)	6.4 (0.2)
A_T_	μM	2483 (47)	2427 (59)	2482 (56)
TCO_2_	μM	2066 (41)	2019 (51)	2056 (44)
HCO_3_^-^	μM	1762 (35)	1723 (44)	1746 (33)
CO_3_^2-^	μM	296 (7)	288 (9)	302 (12)
CO_2_	μM	8.3 (0.2)	8.2 (0.3)	8.0 (0.4)
*p*CO_2_	μatm	300 (8)	296 (9)	289 (7)
NH_4_^+^	μM	0.76 (0.13)	8.33 (0.45)	0.59 (0.07)
NO_3_^-^	μM	0.07 (0.07)	0.46 (0.07)	8.24 (1.31)
*n*		35	34	10

Individual pH values were converted to a H^+^ concentration, allowing a mean pH value to be calculated.

### Light absorption

The effective light absorption coefficients were not significantly different between N-source treatments, nor were the modelled absorption coefficients significantly different to the measured coefficients; with modelled coefficients being only 1 to 3% higher across all N-source treatments (Table 3).

**Table 3:** The mean (± S.E.) measured and modelled effective light absorption coefficients and the relative contribution of each photosynthetic pigment to the total light absorption under the culturing LEDs within *T*. *erythraeum* IMS101, when acclimated to three N-sources (N_2_, NH_4_^+^ and NO_3_^-^), at a target CO_2_ concentration (380 μatm), saturating light intensity (400 μmol photons m^-2^ s^-1^) and optimal temperature (26 °C).

Variables	Units	N_2_	NH_4_^+^	NO_3_^-^
a_eff_^Chl^	m^2^ (g Chl*a*)^-1^	9.9 (0.6)	7.7 (0.9)	8.1 (0.3)
a_eff_^C^	m^2^ (g C)^-1^	0.111 (0.013)	0.154 (0.020)	0.149 (0.006)
a_mod_^Chl^	m^2^ (g Chl*a*)^-1^	10.0 (0.6)	7.8 (0.9)	8.3 (0.3)
a_mod_^C^	m^2^ (g C)^-1^	0.112 (0.013)	0.156 (0.019)	0.154 (0.005)
Chl*a*	%	35.66 (0.41)	36.74 (0.40)	39.18 (2.14)
PPC	%	30.64 (3.19)	27.48 (3.05)	27.44 (2.87)
PUB1	%	2.67 (1.36)^[A]^	5.04 (4.67)	10.11 (2.13)^[B]^
PUB2	%	1.34 (0.28)^[B]^	1.63 (1.04)	0.06 (0.06)^[A]^
PUBx	%	0	0	0
PUB4	%	0.02 (0.02)	0	0
PUB5a	%	0.42 (0.21)	0	0
PUB5b	%	0.24 (0.23)	0	0
PUB5d	%	0.05 (0.03)	0	0
PUBg	%	0.18 (0.18)	0	0
PUBj	%	0.19 (0.19)	0	0
PE1	%	8.10 (3.73)	7.51 (3.70)	3.19 (2.01)
PE2a	%	1.17 (0.75)	0	0
PE2b	%	1.02 (0.72)	0	0
PE3b	%	10.93 (2.28)	13.14 (3.60)	13.03 (0.92)
APC	%	5.45 (1.30)	5.17 (1.34)	5.97 (1.17)
PC1	%	0.94 (0.57)	0.19 (0.19)	0
PC2	%	2.22 (1.15)	3.08 (1.61)	1.02 (0.52)

Light absorption coefficients were spectrally corrected to the culture LEDs and were normalised to a chlorophyll *a* (m^2^ g Chl*a*^-1^) and carbon (m^2^ g C^-1^) basis. Abbreviations; a_eff_^Chl^ and a_eff_^C^ are the measured Chl*a*- and C-specific light absorption coefficients, while a_mod_^Chl^ and a_mod_^C^ are the modelled Chl*a*- and C-specific light absorption coefficients. a_mod_^Chl^ and a_mod_^C^ were constructed from a range of pigment light absorption spectrums (λ = 400–700); comprising chlorophyll *a* (Chl*a*), photoprotective carotenoids (PPC), phycourobilins (PUB1, PUB2, PUBx, PUB4, PUB5a, PUBb, PUB5d, PUB5g and PUB5j), phycoerythrin (PE1, PE2a, PE2b and PE3b), alloplastocyanin (APC) and plastocyanin (PC1 and PC2). Letters in parenthesis indicate significant differences between N-source treatments (One Way ANOVA, Tukey post hoc test; P < .05); where [B] is significantly greater than [A].

**Fig 1:**
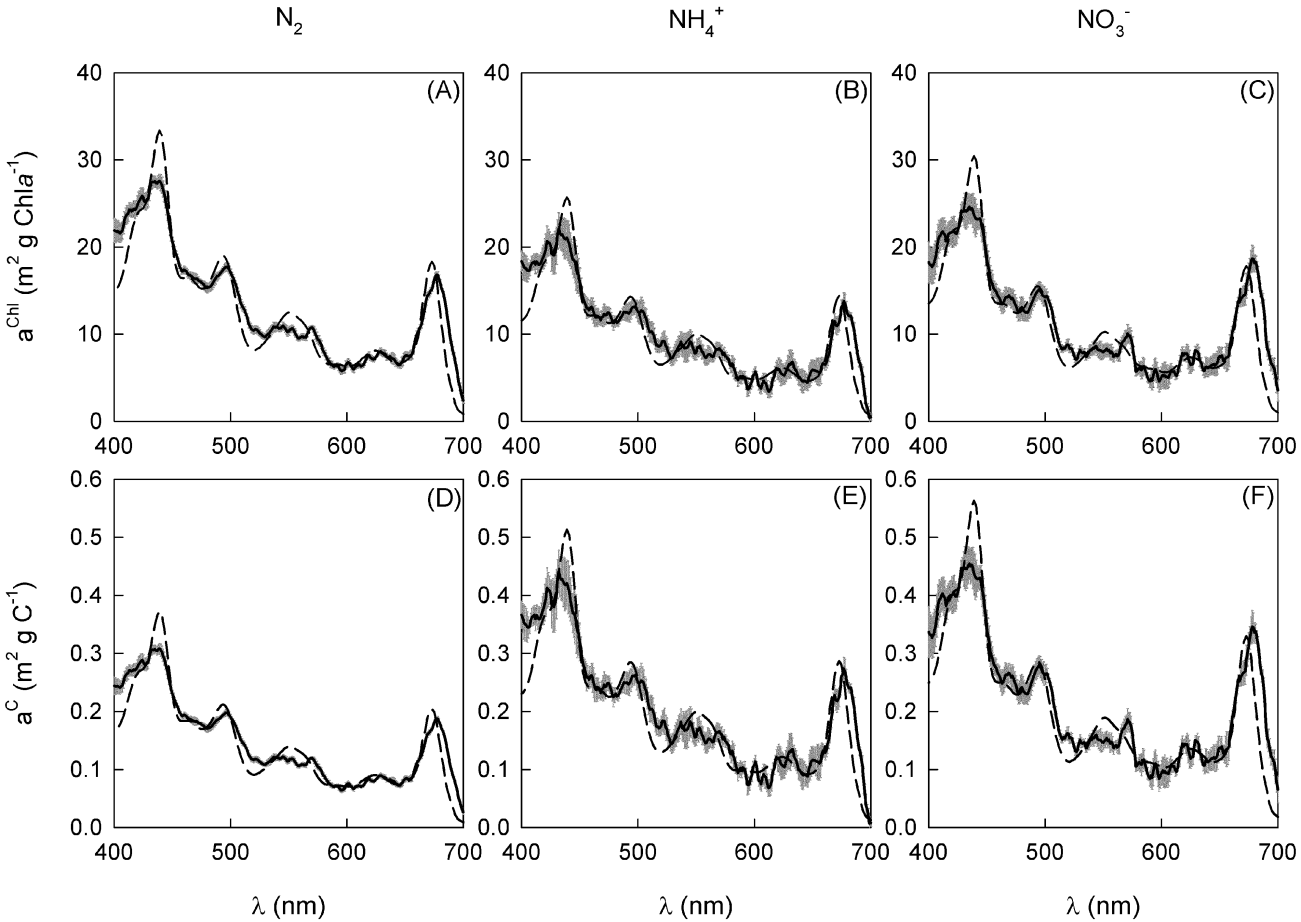
The mean (± S.E.) Chl*a* (a-c) and C-specific (d-f) *in vivo* light absorption spectra for *T*. *erythraeum* IMS101 (*n* = 3). Cultures were acclimated to three N-source treatments (N_2_, NH_4_^+^ and NO_3_^-^), at a target CO_2_ concentration (380 μatm), saturating light intensity (400 μmol photons m^-2^ s^-1^) and optimal temperature (26 °C). The solid black line is the measured light absorption spectra (grey area represents the S.E.) while the dashed line is the modelled light absorption spectra.

*In vivo* light absorption spectra (Fig 1) exhibited peaks at ~ 440 nm (Chl*a*), ~ 490–500 nm (phycourobilin; PUB), ~ 540 and 568 nm (phycoerythrin; PE), ~ 620 nm (phycocyanin; PC), ~ 640 nm (allophycocyanin; APC) and ~ 675 nm (Chl*a*) (Table 3). Chlorophyll *a* and photoprotective carotenoids (PPC) dominated light absorption, together accounting for ~ 65% of the total. PUB1 and PUB2 were the only pigments to exhibit significant differences, where relative to the N_2_ treatment, the contribution of PUB1 to the total light absorption increased by 7.4% whereas PUB2 decreased by 1.3% in the presence of NO_3_^-^ (Table 3).

### Light-dependence of O_2_ exchange

The C-specific maximum rate (E_0m_^C^) and initial slope (α_g_^C^) of light-dependent gross photosynthesis increased with additional N-sources (i.e. NH_4_^+^ and NO_3_^-^) and was highest for the NH_4_^+^ treatment relative to the N_2_ treatment (Table 4). There were also significant effects of additional N-sources on the Chl*a*-specific maximum rate (E_0m_^Chl^) and initial slope of light-dependent gross photosynthesis (α_g_^Chl^) (S1 Table), however the effects were more pronounced when expressed as a C-specific rate, where E_0m_^C^ increased by 143% from the N_2_ to the NH_4_^+^ treatment, while E_0m_^Chl^ increased by only 36%.

**Table 4:** The parameters (± S.E.) of the C-specific light-response curves for gross and net photosynthetic O_2_ evolution of *T*. *erythraeum* IMS101 (*n* = 3).

Parameters	Units	N_2_	NH_4_^+^	NO_3_^-^
Gross O_2_ evolution				
E_0m_^C^	mmol O_2_ (g C)^-1^ h^-1^	6.05 (0.37)^[A]^	14.71 (1.20)^[C]^	10.98 (0.33)^[B]^
E_k_	μmol photons m^-2^ s^-1^	238 (55)	227 (44)	255 (55)
α_g_^C^	μmol O_2_ (g C)^-1^ h^-1^ (μmol photons m^-2^ s^-1^)^-1^	27.9 (5.3)^[A]^	67.7 (8.2)^[B]^	49.8 (15.1)
ɸ_mgross_	mol O_2_ (mol photons)^-1^	0.07 (0.01)^[A]^	0.12 (0.01)^[B]^	0.09 (0.03)
E_0_:N_fix_	mol O_2_ (mol N_2_)^-1^	31 (4)^[A]^	289 (32)^[B]^	185 (57)^[B]^
Net Photosynthesis				
P_netm_^C^	mmol O_2_ (g C)^-1^ h^-1^	3.75 (0.27)^[A]^	11.48 (1.56)^[B]^	9.59 (0.37)^[B]^
E_k_	μmol photons m^-2^ s^-1^	250 (69)	277 (8)	220 (37)
α_n_^C^	μmol O_2_ (g C)^-1^ h^-1^ (μmol photons m^-2^ s^-1^)^-1^	16.8 (3.4)^[A]^	41.5 (5.8)^[B]^	46.1 (8.0)^[B]^
R_d_^C^	mmol O_2_ (g C)^-1^ h^-1^	-1.63 (0.19)	-1.53 (0.28)	-1.16 (0.76)
ɸ_mnet_	mol O_2_ (mol photons)^-1^	0.04 (0.01)^[A]^	0.08 (0.02)^[B]^	0.09 (0.01)^[B]^
P_net_:N_fix_	mol O_2_ (mol N_2_)^-1^	18 (1)^[A]^	207 (29)^[B]^	163 (36)^[B]^
Gross (*x*) vs. Net (*y*)				
slope	Dimensionless	0.60 (0.02)^[A]^	0.82 (0.03)^[B]^	0.83 (0.01)^[B]^

Abbreviations; E_0m_^C^, the C-specific maximum gross O_2_ evolution rate; P_netm_^C^, the C-specific maximum net O_2_ evolution rate; E_k_, the light saturation parameter; α_g_^C^ and α_n_^C^ are the C-specific initial slopes the light response curve for net and gross photosynthesis; ɸ_mgross_ and ɸ_mnet_ are the maximum quantum efficiencies of gross and net O_2_ evolution; R_d_^C^, the C-specific dark respiration rate; slope, the gradient of the regression between P_net_^C^ and E_0_^C^; E_0_:N_fix_ and P_net_:N_fix_, the ratio of gross and net photosynthesis to N_2_ fixation, where rates of E_0_ and P_net_ were calculated at 400 μmol photons m^-2^ s^-1^, matching to light intensity of the N_2_ fixation incubations; slope, the gradient of the regression between P_net_^C^ and E_0_^C^. The *r*^2^ values of all curve fits were > 0.982. Letters in parenthesis indicate significant differences between CO_2_ treatments (One Way ANOVA, Tukey post hoc test; P < .05); where [B] is significantly greater than [A] and [C] is significantly greater than [B] and [A].

The light saturation parameter (E_k_ = E_0m_^C^/α_g_^C^) of gross O_2_ evolution did not vary significantly between N-source treatments (Table 4) and was due to covariation of α_g_^C^ and E_0m_^C^. The maximum quantum efficiency of gross O_2_ evolution (ɸ_mgross_ = α_g_^C^/a_eff_^C^) increased significantly by 76% from the N_2_ to NH_4_^+^ treatment (Table 4) and was due to the relatively constant a_eff_^C^ and the significant increase in α_g_^C^.

Carbon-specific dark respiration rates (R_d_^C^) varied by ~ 24% and were slightly higher for the N_2_ and NH_4_^+^ treatments than the NO_3_^-^ treatment (Table 4). Light-saturated net O_2_ evolution rates (P_netm_^C^) approximately trebled and more than doubled from the N_2_ treatment to the NH_4_^+^ and NO_3_^-^ treatments respectively (Table 4); with the initial slope (α_n_^C^) showing a similar pattern to P_netm_^C^. This increase in α_n_^C^ for the NH_4_^+^ and NO_3_^-^ treatments resulted in the maximum quantum efficiency of net O_2_ evolution (ɸ_mnet_ = α_n_^C^/a_eff_^C^) increasing significantly by 86% and 100% respectively, relative to the N_2_ treatment (Table 4). The light saturation parameter (E_k_ = P_netm_^C^/α_g_^C^) for net O_2_ evolution did not vary significantly between N-source treatments (Table 4).

**Fig 2:**
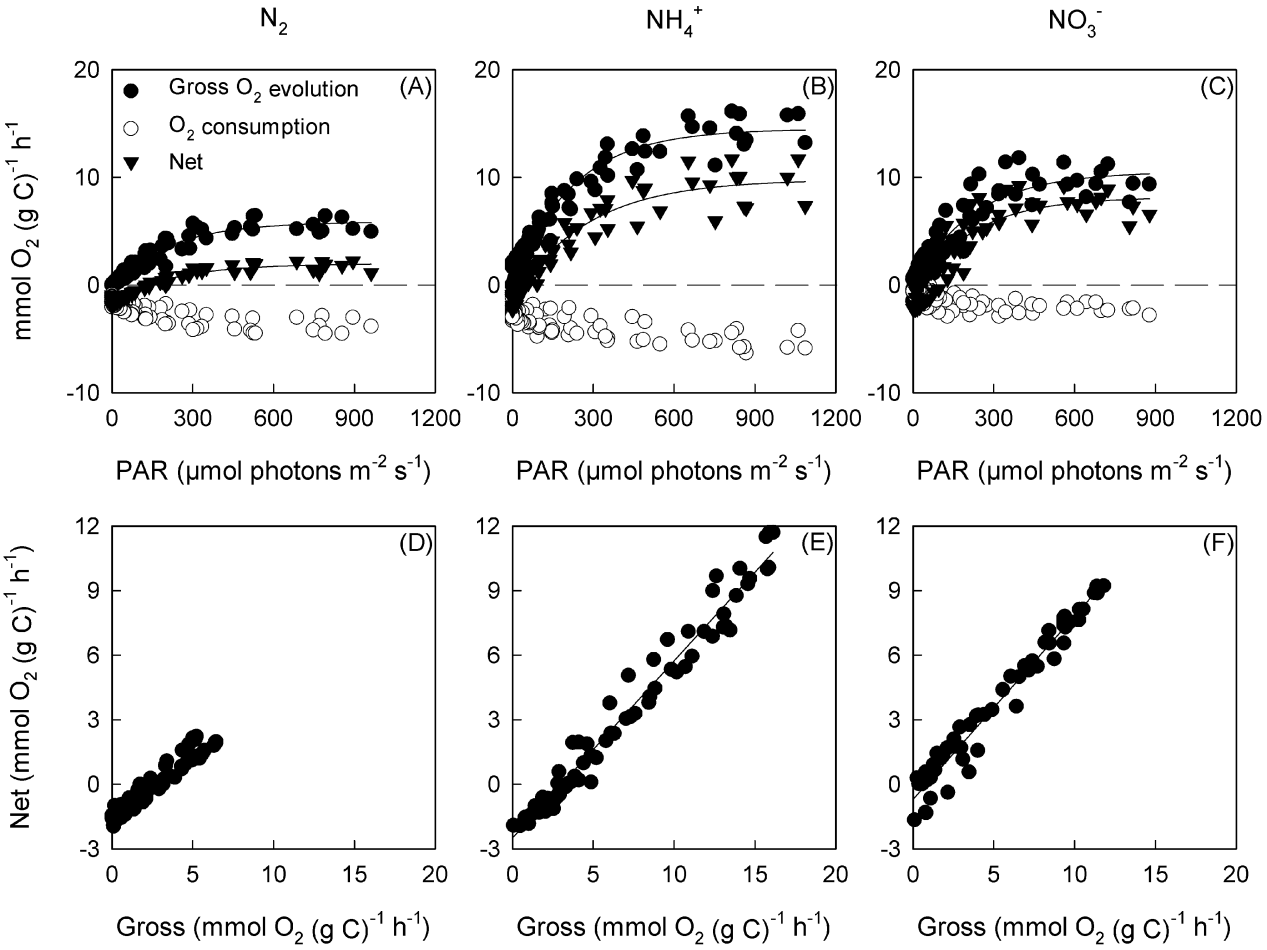
The C-specific light response curves for gross O_2_ evolution, O_2_ consumption, net photosynthesis (*n* = 3) (a-c) and the relationship between gross and net O_2_ evolution (d-f) for *T*. *erythraeum* IMS101. Cultures were acclimated to three N-sources (N_2_, NH_4_^+^ and NO_3_^-^), at a target CO_2_ concentration (380 μatm), saturating light intensity (400 μmol photons m^-2^ s^-1^) and optimal temperature (26 °C). Chl*a*-specific light response curves are shown in S4 Fig, while the light response curves for individual replicates are shown in S6–S8 Fig.

The relationship between net and gross O_2_ evolution was linear (Fig 2D–2F), with the slope increasing by approximately 40% when cultured in the presence of an additional N-source (Table 4). This linear relationship suggests that light-dependent O_2_ consumption (U_0_^C^) was a constant proportion of gross O_2_ evolution (E_0_^C^) and was independent of light intensity for all N-source treatments. Subtracting the slope from unity gave the ratio of light-driven U_0_^C^ to E_0_^C^, which was significantly lower for the N_2_ treatment.

The ratio of gross photosynthesis (E_0_) to N_2_ fixation increased 9-fold and 6-fold for the NH_4_^+^ and NO_3_^-^ treatments relative to the N_2_ treatment. In addition, the ratio of net photosynthesis (P_net_) to N_2_ fixation was 12-fold and 7-fold higher for the NH_4_^+^ and NO_3_^-^ treatments relative to the N_2_ treatment (Table 4).

### Light-dependence of PSII electron transport

**Fig 3:**
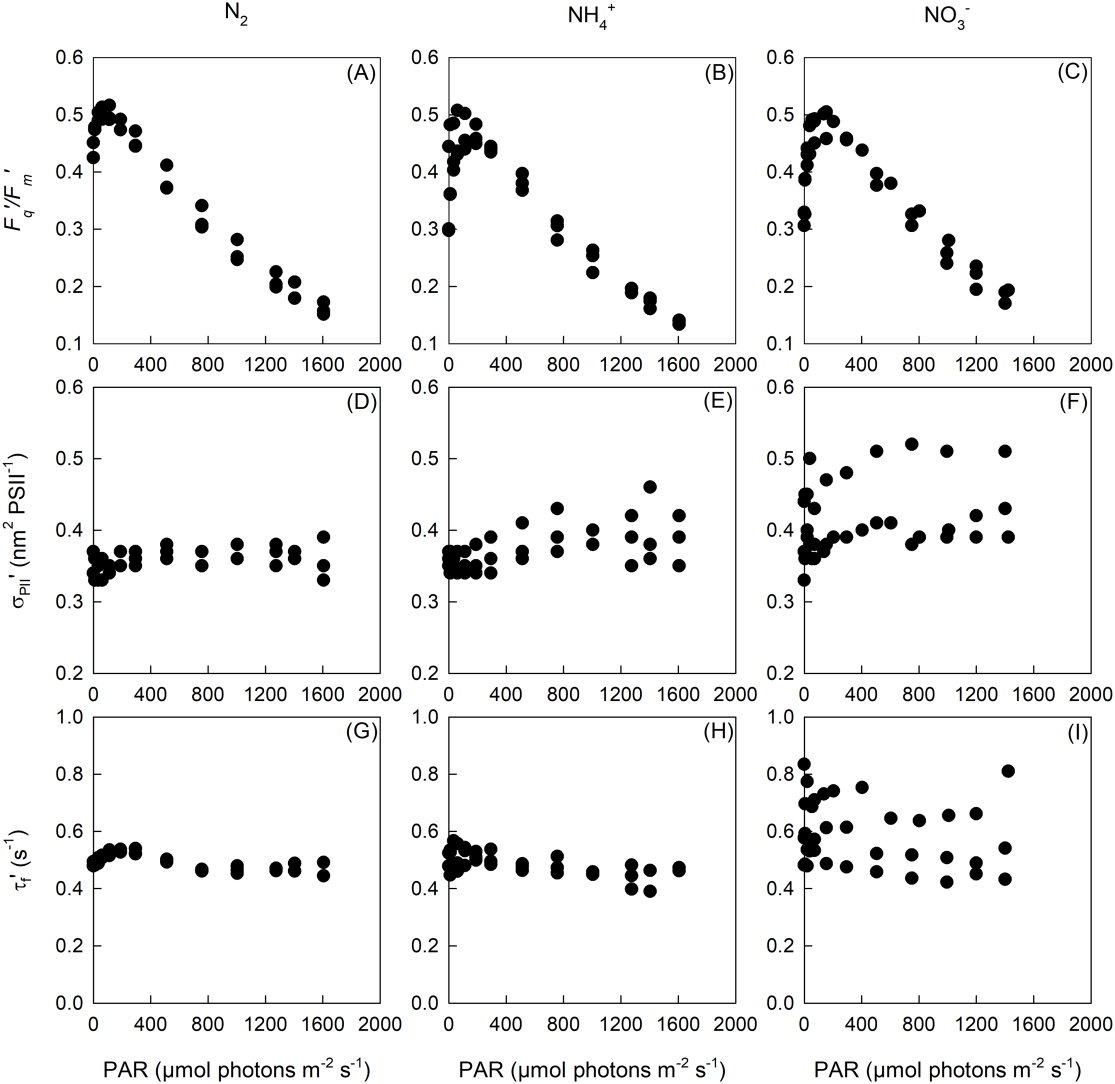
The operating efficiency of PSII photochemistry (*F*_*q*_ʹ/*F*_*m*_ʹ) (a-c), light absorption cross-section of PSII photochemistry (σ_PII_ʹ) (d-f) and average time constant for the re-opening of a closed PSII reaction centres (τ_f_ʹ) (g-i) across a range of actinic light intensities for *T*. *erythraeum* IMS101 (*n* = 3). Cultures were acclimated to three N-sources (N_2_, NH_4_^+^ and NO_3_^-^), at a target CO_2_ concentration (380 μatm), saturating light intensity (400 μmol photons m^-2^ s^-1^) and optimal temperature (26 °C).

The operating efficiency of PSII photochemistry (*F*_*q*_*'*/*F*_*m*_*ʹ*) increased at low light intensities, reaching a maximum at ~ 110 to 130 μmol photons m^-2^ s^-1^, before decreasing significantly with increasing light intensity (Fig 3). The light saturation parameter (E_k_) and the light at which ETR was maximal (E_opt_) were significantly higher for the N_2_ treatment than the NH_4_^+^ treatment. Conversely, the light inhibition parameter (E_p_), absorption cross-section of PSII photochemistry (σ_PII_) and the time constant for the re-opening of a closed PSII reaction centre (τ_f_) in the dark-adapted state were not significantly different between N-source treatments. Furthermore, both σ_PII_ʹ and τ_f_ʹ exhibited no light-dependency, remaining relatively constant across the entire range of actinic light intensities (Fig 3, Table 5).

**Table 5:** The parameters (± S.E.) of the fluorescence light-response curves (FLCs) of *T*. *erythraeum* IMS101 (*n* = 3).

Parameters	Units	N_2_	NH_4_^+^	NO_3_^-^
ETR_m_^C^	mmol e^-^ (g C)^-1^ h^-1^	62.5 (16.7)	70.3 (14.4)	91.0 (4.9)
E_k_	μmol photons m^-2^ s^-1^	465 (8)^[B]^	421 (3)^[A]^	447 (20)
α_ETR_^C^	mmol e^-^ (g C)^-1^ h^-1^ (μmol photons m^-2^ s^-1^)^-1^	0.133 (0.033)	0.167 (0.033)	0.200 (0.003)
β_ETR_^C^	mmol e^-^ (g C)^-1^ h^-1^ (μmol photons m^-2^ s^-1^)^-1^	5081 (55)^[A]^	5577 (55)^[C]^	5332 (14)^[B]^
E_opt_	μmol photons m^-2^ s^-1^	1263 (22)^[B]^	1144 (3)^[A]^	1216 (54)
E_p_	μmol photons m^-2^ s^-1^	0.99 (0.11)	0.67 (0.08)	0.83 (0.09)
*F*_*v*_/*F*_*m*_	Dimensionless	0.44 (0.01)	0.35 (0.05)	0.32 (0.01)
σ_PII_	nm^2^ PSII^-1^	0.353 (0.009)	0.367 (0.003)	0.380 (0.032)
τ_f_	s^-1^	489 (5)	494 (15)	631 (105)
Φ_em_	mol e^-^ (mol O_2_)^-1^	10.5 (1.1)^[B]^	5.7 (1.1)^[A]^	7.9 (0.5)
Φ_eα_	mol e^-^ (mol O_2_)^-1^	5.2 (0.8)	2.8 (0.2)	4.5 (1.0)

Abbreviations; ETR_m_^C^, the C-specific maximum electron transport rate; α_ETR_^C^, the C-specific initial slope of the electron transport rate light response curve; β_ETR_^C^, the C-specific light saturated slope of the electron transport rate light response curve; E_k_, the light saturation parameter; E_opt_, the light at which ETR is maximal; E_p_, the light inhibition parameter; *F*_*v*_/*F*_*m*_, the maximum photochemical efficiency of PSII in the dark-adapted state; σ_PII_, the absorption cross-section of PSII photochemistry in the dark-adapted state; τ_f_, the average time constant for the re-opening of a closed PSII reaction centre in the dark-adapted state; Φ_em_, the light saturated ratio of PSII electron transport to gross O_2_ evolution; Φ_eα_, the light limited ratio of PSII electron transport to gross O_2_ evolution. The *r*^2^ values of all curve fits were > 0.977. Letters in parenthesis indicate significant differences between N-source treatments (One Way ANOVA, Tukey post hoc test; P < .05); where [B] is significantly greater than [A] and [C] is significantly greater than [B] and [A].

**Fig 4:**
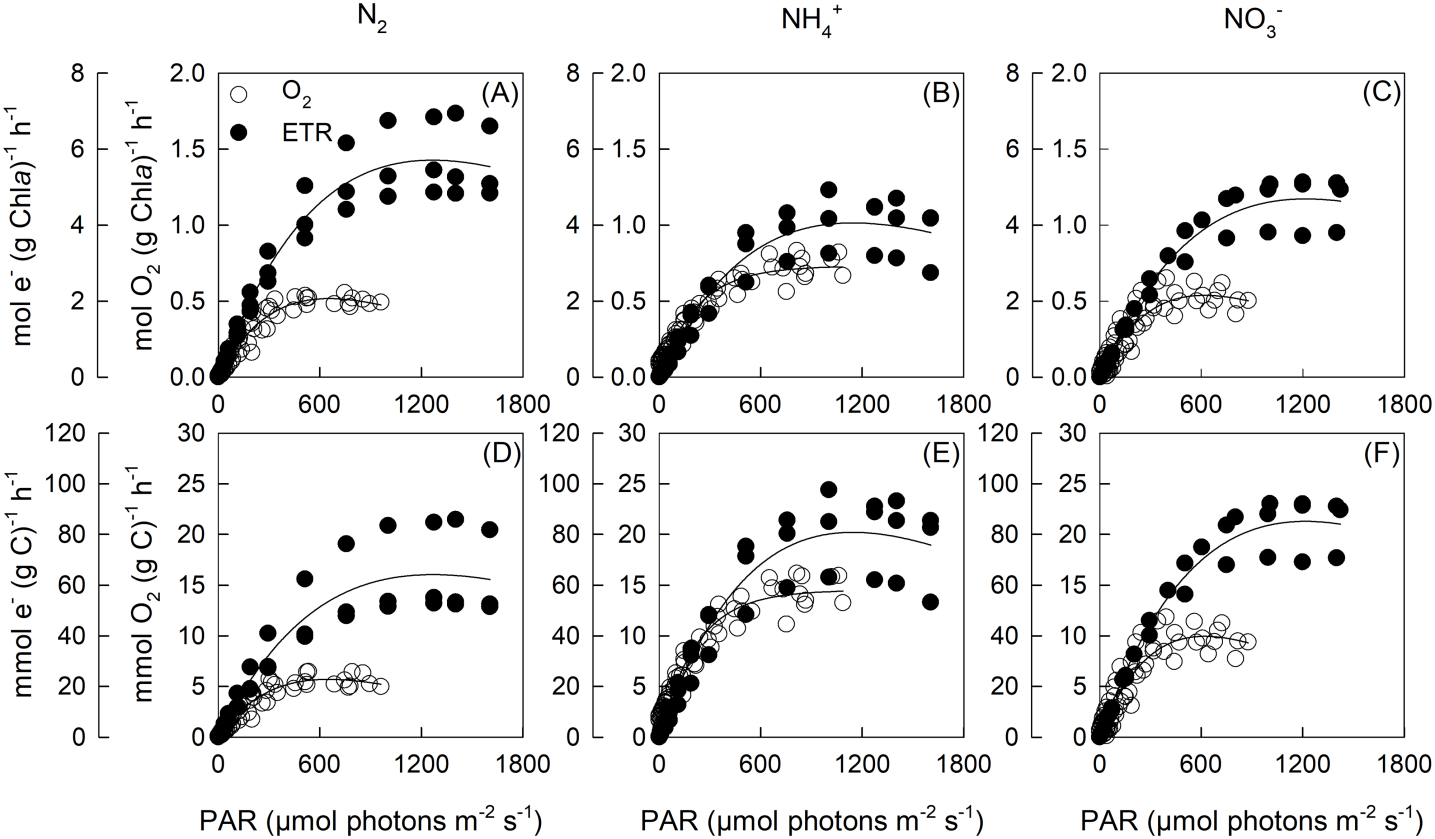
Concurrent Chl*a* (a-c) and C-specific (d-f) gross O_2_ evolution rates and PSII electron transport rates (ETR) for *T*. *erythraeum* IMS101 (*n* = 3). Cultures were acclimated to three N-sources (N_2_, NH_4_^+^ and NO_3_^-^), at a target CO_2_ concentration (380 μatm), saturating light intensity (400 μmol photons m^-2^ s^-1^) and optimal temperature (26 °C).

The light intensity at which ETR was maximal (E_opt_) was significantly lower (by ~ 120 μmol photons m^-2^ s^-1^) for the NH_4_^+^ treatment relative to the N_2_ treatment (Fig 4). The Chl*a* and C-specific maximum electron transport rate and initial slope (α_ETR_) of the ETR-light curves were not significantly different between N-source treatments (Table 5, S2 Table). In contrast, the light-saturated photoinhibition slopes (β_ETR_) were significantly different, with β increasing by 5% and 10% for the NO_3_^-^ and NH_4_^+^ treatments, relative to the N_2_ treatment (Table 5).

The ratio of PSII electron transport to gross O_2_ evolution under light-limitation (Φ_eα_) was ~ 4 and did not vary significantly between N-source treatments. Light saturated ratios (Φ_em_) increased relative to Φ_eα_ for all N-source treatments, with the N_2_ treatment being 46% and 35% higher than the NH_4_^+^ and NO_3_^-^ treatments, respectively (Table 5).

## Discussion

### Effect of acclimation to variation of N-sources on growth rates and elemental stoichiometry

Growth rates achieved under diazotrophic conditions were similar to most previous studies [23, 43–45], as was the increase in growth rate observed under non-diazotrophic conditions [23, 43], which we attribute to the lowered demand of NADPH and ATP for nitrogenase activity, where NADPH and ATP could be re-directed to CO_2_ fixation and/or biosynthesis. Our data shows that at saturating light intensity, the energetic cost of diazotrophy constrains *Trichodesmium* growth by ~ 13%. However, in a natural system, potential changes to inorganic carbon chemistry (influencing the activity of the carbon concentrating mechanism (CCM)), temperature (influencing enzyme activity), or other key nutrients (i.e. Fe, P), all of which were controlled in our experiments, will almost certainly influence this estimate.

The decrease in C:N, C:P and N:P under non-diazotrophic conditions is consistent with previous findings [43]. The high C:N under diazotrophic conditions may be due to accumulation of stored glycogen, whereas the decrease in C:N under non-diazotrophic conditions is likely due to high cellular N concentrations, likely due to the luxury uptake of NH_4_^+^ and NO_3_^-^, where surplus N is stored within cyanophycin granules [26]. Given the concurrent decrease in C:N, C:P and N:P under non-diazotrophic conditions, it is likely that utilising NH_4_^+^ or NO_3_^-^ as a N-source enables *Trichodesmium* cells of low carbon biomass to maintain a Chl*a* concentration comparable to diazotrophic conditions. This is supported by previous observation made by Eichner *et al*. [43] and is also reflected by the higher Chl*a*:C yet comparable Chl*a*:N ratios for NH_4_^+^ or NO_3_^-^ treatments.

Growing evidence points towards nitrogenase being expressed in subsets of cells within filaments, called diazocytes [21, 25]. To date, no translocation transport mechanisms for N compounds have been observed in *Trichodesmium*, leading to suggestions that diazocytes release N into the external medium for use by neighbouring cells [25, 46]. This is partially supported by observations of *Trichodesmium* exhibiting a high capacity for NH_4_^+^ uptake during active N_2_ fixation [17, 18]. While such mechanisms may exist, we did not observe significant concentrations of dissolved inorganic NO_3_^-^ or NH_4_^+^ in the medium of our control treatment.

### Effect of acclimation to different N-sources on gross photosynthesis

We show an effect of N-source on C-specific light saturated gross O_2_ evolution rates. The more than two-fold increase in the maximum O_2_ evolution rate and initial slope when *T*. *erythraeum* IMS101 was grown on NH_4_^+^ or NO_3_^-^ than when growing diazotrophically was largely due to differences in the ratio of Chl*a*:C as chlorophyll *a*-specific photosynthetic parameters varied by only 36% between N_2_ and NH_4_^+^ treatments.

The increase of C-specific gross O_2_ evolution rates when *Trichodesmium* is supplied with NH_4_^+^ or NO_3_^-^ may be due to an increase in the maximum rate of CO_2_ fixation and/or to an increase in PSII concentration. Previous studies report high PSI:PSII ratios under diazotrophic conditions (ranging between 1.3 to 4) [47–51], which would allow cyclic photophosphorylation in diazocytes to provide most of the ATP required for N_2_ fixation, with glycolysis and the Kreb’s cycle providing the required reducing equivalent. It may be that under non-diazotrophic conditions and with lower nitrogenase activity, *Trichodesmium* enhances linear electron transport to increase NADPH production; a pathway that generates more evolved O_2_.

### Effect of acclimation to different N-sources on N_2_ fixation

Nitrogenase activity declined significantly by 81–84% when *Trichodesmium* was cultured in the presence of an additional N-source. Despite being cultured under N-replete concentrations, *Trichodesmium* cells in the NH_4_^+^ and NO_3_^-^ treatments exhibited a baseline rate of N_2_ fixation. Similarly, Milligan *et al*. [52] reported a ~ 85% decrease when *Trichodesmium* was cultured in 100 μM of NO_3_^-^ for 2 weeks and Holl and Montoya [44] reported a 66% decrease when cultured in 20 μM of NO_3_^-^, accrediting 8% of total N assimilation to diazotrophy despite the presence of additional N-sources. Maintaining the capability to perform N_2_ fixation under non-diazotrophic conditions, albeit at a reduced rate, could reflect *Trichodesmium’s* natural environment and act a potential safeguard mechanism to variable light and nutrient regimes.

Noting that 16 moles of ATP are consumed per mole of N_2_ fixed (Eq 1) and that 2.56 moles of ATP can be produced per mole of O_2_ evolved by photophosphorylation linked LPET [53], we calculated that *T*. *erythraeum* IMS101 may use 20% of the ATP that could be generated from gross O_2_ evolution to support the observed N_2_ fixation rate during diazotrophic growth:

$$\frac{N_{fix}}{E_{0}}\;=\;\frac{{1\;mol\;N}_{2}}{{31\;mol\;O}_{2}}∙\frac{{1\;mol\;O}_{2}}{2.54\;mol\;ATP}∙\frac{16\;mol\;ATP}{{1\;mol\;N}_{2}}\;=\;0.2$$ (24)

This proportion decreases to 2% and 4% for the NO_3_^-^ and NH_4_^+^ treatments, respectively, where the ratio of E_0_:N_fix_ increases to 289 for the NO_3_^-^ treatment and 185 in the NH_4_^+^ treatment, versus 31 in the N_2_ treatment (Table 4).

Studies on natural populations of *Trichodesmium* spp. have shown that the addition of NO_3_^-^ (100 μM) in the morning can cause a gradual decrease of N_2_ fixation over the photic period [22]. Further studies have also shown that addition of glutamine (10 μM) immediately decreases N_2_ fixation rates, indicating a direct effect on enzyme activity as opposed to enzyme synthesis [54]. These observations have been accredited to accumulation of N-containing metabolites acting as potential inhibitors to the specific activity rather than abundance of nitrogenase [22, 54].

It is well known that intracellular nitrogen pools have a role in regulating nitrogenase activity in diazotrophs [55, 56]. Dinitrogenase reductase catalyses the reduction of N_2_ to NH_4_^+^, which is assimilated into glutamine (gln) and then into glutamate (glu) via the glutamine synthetase (GS, EC 6.3.1.2)/glutamate synthase (GOGAT) pathway [54]. The intracellular pools of NH_4_^+^, glu and gln have been identified as important feedback regulators of N uptake and metabolism, with GS activity in *Trichodesmium* being sensitive to both intra- and extracellular N concentrations [55]. It could be hypothesised that the activity of nitrogenase is influenced by internally recycled N (e.g. NH_4_^+^ and gln), while the synthesis of nitrogenase is influenced by newly assimilated N (e.g. NO_3_^-^).

### Effect of acclimation to different N-sources on light-stimulated O_2_ consumption and the relationship between net and gross O_2_ evolution

Net photosynthesis was significantly lower for the N_2_ treatment than for the NH_4_^+^ and NO_3_^-^ treatments. Despite slight variations in E_0_^C^, the difference in net photosynthesis was principally driven by O_2_ consumption. Approximately 68%, 32% and 29% (N_2_, NH_4_^+^ and NO_3_^-^, respectively) of E_0_^C^ was consumed by O_2_ consuming processes, which is comparable to previous observations [43, 52].

Several processes demand ATP in excess of the ATP:NADPH produced through linear photophosphorylation; two most notably being N_2_ fixation and the operation of the CCM [57]. In this study, the carbon chemistry of all cultures was closely regulated to ensure that variation in O_2_ consumption and net photosynthesis was due to the N-source treatments only. Linearity between gross O_2_ evolution (E_0_) and O_2_ consumption was observed across all N-source treatments, suggesting that light-dependent O_2_ consumption is linked to balancing ATP to NADPH production, as opposed to serving as a mechanism to dissipate excitation energy.

Diazotrophic cells consume more O_2_ per evolved O_2_ across the entire range of actinic light intensities than the NH_4_^+^ and NO_3_^-^ treatments. This suggests a higher rate of water-water cycling due to either Mehler activity or operation of plastoquinone terminal oxidase when N_2_ is being fixed. To maintain a sufficient supply of ATP relative to NADPH, *Trichodesmium* may utilise pseudocyclic photophosphorylation linked to the Mehler reaction to augment the ATP generated by linear electron transfer from water to NADP^+^ in addition to ATP produced by cyclic electron flow around PSI.

Measurements of O_2_ evolution, ETR and N_2_ fixation were all made at one time of day (4 to 6 hours into the photo-phase of a 12:12 L:D cycle) and as such cannot be extrapolated to a diel response given the reports of temporal separation of photosynthesis and N_2_ fixation in *Trichodesmium* [21].

### Effect of acclimation to different N-sources on electron transport rates and photophysiology

Like Eichner *et al*. [43], we observed a negligible effect of N-source on many photo-physiological parameters, including *F*_*q*_ʹ/*F*_*m*_ʹ, σ_PII_ and τ_f_. *Trichodesmium* exhibited a light response typical for most cyanobacteria, where the dark-adapted photochemical yield is significantly affected by respiratory electron flow [58]. This results from a proportion of PSII reaction centres remaining in a closed state despite being in the dark and is imposed by a reduction in the plastoquinone (PQ) pool, which prevents the oxidation of Q_A_^-^. Moving from darkness to a low light intensity increases the electron flux through PSI, alleviates the bottleneck of electron transport through the Cyt *b6f* complex, thereby increasing *F*_*q*_ʹ/*F*_*m*_ʹ and decreasing the re-oxidation time of Q_A_^-^. Addition factors such as higher downregulation under dark-adapted conditions may also contribute to the increase in *F*_*q*_ʹ/*F*_*m*_ʹ under low light intensities.

### Ratio of electron transport to gross O_2_ evolution

Electrons are transferred from PSII (where O_2_ is evolved) to an intermediate plastoquinone pool and eventually to ferredoxin to produce NADPH [59]. A minimum of four moles of electrons are transported through PSII for each mole of O_2_ evolved at PSII. Most higher plants exhibit a linear correlation between gross O_2_ evolution and electron transport rate [60]. In microalgae, this relationship is often ambiguous, especially at high light intensities where the relationship can become non-linear [61, 62].

Here we show that at low light intensities, the ratio of PSII electron transport to gross O_2_ evolution (Φ_eα_) is close to a 4:1 ratio for all N-sources treatments. However, when light intensities exceed 150 μmol photons m^-2^ s^-1^, Φ_e_ declines as ETR saturates at a higher light intensity (~ 900 μmol photons m^-2^ s^-1^) than E_0_ (~ 400 μmol photons m^-2^ s^-1^). Similar responses have been reported for diatoms [63], microalgae [64] and the Baltic cyanobacteria, *Nostoc* [65]. Few studies have measured O_2_ production rates in *Trichodesmium* [47, 66] and to our knowledge none have reported concurrent PSII electron transport rates.

Interestingly, we calculated a higher Φ_e_ for the N_2_ cultures than for the NH_4_^+^ and NO_3_^-^ cultures, irrespective of using the light-limited or -saturated rates. This may be due to overestimating the proportion of light absorbed by PSII in the non-diazotrophic growth conditions (i.e. NH_4_^+^ and NO_3_^-^) relative to the diazotrophic condition. Here we assumed that 50% of absorbed light was directed to PSII reaction centres and 50% to PSI reaction centres (i.e. FAQ_PII_ of 0.5). It’s likely that FAQ_PII_ was overestimated for diazotrophic treatment (i.e. N_2_) which may have had a higher ratio of PSI:PSII to support significant rates of cyclic photophosphorylation. In addition, non-diazotrophic cells may undergo more pronounced state transitions with phycobilin proteins being redistributed between PSII and PSI. Finally, a Φ_e_ > 4 could be accredited to cyclic electron flow around PSII, which may act a mechanism to dissipate excess excitation energy under high light [67].

### Implications for future oligotrophic oceans

In N-limited regions of the oligotrophic open ocean, diazotrophy provides a competitive advantage by allowing cells to access N_2_ as an N-source against faster growing phytoplankton that rely on fixed N. Current ocean models predict a poleward shift in the 20 °C isotherm which could extend *Trichodesmium’s* niche into higher latitudes. On a global scale, this niche expansion is driven by increased SSTs; however, on regional scales persistence in an area may be dictated by *Trichodesmium’s* response to fluctuating nutrient regimes.

At the surface in oligotrophic waters, *Trichodesmium* is unlikely to encounter NO_2_^-^, NO_3_^-^ or NH_4_^+^ concentrations in excess of 0.1 μM [68], except during mixing events. While *Trichodesmium* is commonly observed in the upper meters of the water column [69], observations have been recorded down to 200 m depth [70]. Thus, *Trichodesmium* colonies and free trichomes are able to migrate to the nutricline [30, 31]. Such vertical migration has been suggested to allow luxury uptake of phosphates before colonies return to the surface. In addition to encountering phosphates, *Trichodesmium* will also encounter high concentrations of NO_3_^-^ in the nutricline. As such, NO_3_^-^ uptake is likely at these greater depths or at the surface after a mixing event.

Mulholland *et al*. [17] reported significant NO_3_^-^ uptake rates with the addition of 1 μM NO_3_^-^ to the growth media. Furthermore, Karl *et al*. [30] showed that concentrations of dissolved NH_4_^+^ reached 1.5 μM L^-1^ and dissolved organic N (DON) reaching 13 μM L^-1^ during a natural bloom of *Trichodesmium* spp. in the North Pacific gyre. These concentrations are far greater than typical oceanic N pools and could therefore be high enough to inhibit N_2_ fixation rates [44]. It’s therefore possible that *Trichodesmium* colonies at depth may be utilising more combined N-sources than the blooms frequently measured on the surface. The energy and reductant conserved through utilising additional N-sources could significantly enhance *Trichodesmium’s* productivity and growth which could have major implications for biogeochemical cycles.

Our results indicate the need to seek more information on the potential for natural populations of *Trichodesmium* to uptake fixed N-sources (e.g. NO_3_^-^, NH_4_^+^, labile dissolved organic nitrogen (DON)) at concentrations that migrating colonies or trichomes experience in the nutricline or that are encountered transiently after deep mixing events. The potential significance of *Trichodesmium* assimilating fixed N is indicated by a modelling study by McGillicuddy [33] which concluded that to obtain realistic simulations of biomass and export production *Trichodesmium* populations in the North Atlantic must utilise fixed N. Specifically, this study indicated that 15–20% of the N quota of *Trichodesmium* could be due to uptake of NO_3_^-^ and NH_4_^+^. Furthermore, although uptake of NO_3_^-^, NH_4_^+^ or DON will decrease N_2_ fixation rates in the short-term, as these N-sources are depleted over longer time periods, the increase in *Trichodesmium* biomass may lead to increased N_2_ fixation and greater competition for other nutrients including Fe and P.

## Supporting information

S1 Fig The relative fluorescence excitation spectra of *T*. *erythraeum* IMS101 (Bold solid line) and the relative emission spectra of the Iso Light 400 LED (white) block used for O_2_ evolution incubations (Solid line), FRRf LED (blue) used for the saturating flashlets (Long-Dashed line), FastAct LED (white) used for the actinic light source (Short-dashed line) and the culturing LED (white) (Dotted line). (A) The fluorescence excitation was measured on a 2 mL concentrated sample treated with 20 μM DCMU (final concentration) [71]. *Trichodesmium* cells were acclimated to 150 μmol photons m^-2^ s^-1^ on a 14:10 light:dark cycle, 26 °C and ambient CO_2_. The sample was measured using a FluorWin fluorometer scanning between 400 to 715 nm at a 1 nm resolution, with the monochromator on the detector set to 730 nm emission [72]. Spectral correction factors were calculated using the FastPro8. (B) An example of an *in vivo* light absorption spectra of *T*. *erythraeum* IMS101 when spectrally corrected to the Culture, MIMS or FRRf LED spectra.
(TIF)

S2 Fig Reconstructed light absorption spectra of eighteen key photosynthetic pigments present within *T*. *erythraeum* IMS101. (A) The light absorption spectra of chlorophyll *a* (Chl*a*), photoprotectant carotenoid (PPC), phycoerythrin (PE), plastocyanin (PC) and alloplastocyanin (APC) pigments. (B) The light absorption spectra of phycourobilin (PUB) pigments. Each pigment spectra was normalised to the maximum peak (λ = 400–700 nm).
(TIF)

S3 Fig Inorganic carbon chemistry (Ci) of *T*. *erythraeum* IMS101 cultures, measured at 2-hour intervals over the light period. The pH and TCO_2_ was measured directly, while the *p*CO_2_ concentrations were calculated via *CO2SYS* using the same constants as described in Boatman *et al*. [45] and S1 File.
(TIF)

S4 Fig Chl*a*-specific light response curves for gross O_2_ evolution, O_2_ consumption, net photosynthesis (*n* = 3) (A-C) and the relationship between gross and net O_2_ evolution (D-F) for *T*. *erythraeum* IMS101. Cultures were acclimated to three N-sources (N_2_, NH_4_^+^ and NO_3_^-^), at a target CO_2_ concentration (380 μatm), saturating light intensity (400 μmol photons m^-2^ s^-1^) and optimal temperature (26 °C).
(TIF)

S5 Fig Percentage of the modelled *in vivo* light absorption (a_mod_) associated to each photosynthetic pigment (λ = 400–700) for *T*. *erythraeum* IMS101. Cultures were acclimated to three N-sources (N_2_, NH_4_^+^ and NO_3_^-^), at a target CO_2_ concentration (380 μatm), saturating light intensity (400 μmol photons m^-2^ s^-1^) and optimal temperature (26 °C). Pigments include chlorophyll *a* (Chl*a*), photoprotectant carotenoid (PPC), phycourobilins (PUB1, PUB2, PUBx, PUB4, PUB5a, PUBb, PUB5d, PUB5g and PUB5j), phycoerythrin (PE1, PE2a, PE2b and PE3b), alloplastocyanin (APC) and plastocyanin (PC1 and PC2).
(TIF)

S6 Fig Chl*a* and C-specific light response curves for gross O_2_ evolution, O_2_ consumption, net photosynthesis (*n* = 3) (A-C) and the relationship between gross and net O_2_ evolution (D-F) for *T*. *erythraeum* IMS101. Cultures were acclimated to N_2_-only, at a target CO_2_ concentration (380 μatm), saturating light intensity (400 μmol photons m^-2^ s^-1^) and optimal temperature (26 °C).
(TIF)

S7 Fig Chl*a* and C-specific light response curves for gross O_2_ evolution, O_2_ consumption, net photosynthesis (*n* = 3) (A-C) and the relationship between gross and net O_2_ evolution (D-F) for *T*. *erythraeum* IMS101. Cultures were acclimated to a replete NH_4_^+^ concentration, at a target CO_2_ concentration (380 μatm), saturating light intensity (400 μmol photons m^-2^ s^-1^) and optimal temperature (26 °C).
(TIF)

S8 Fig Chl*a* and C-specific light response curves for gross O_2_ evolution, O_2_ consumption, net photosynthesis (*n* = 3) (A-C) and the relationship between gross and net O_2_ evolution (D-F) for *T*. *erythraeum* IMS101. Cultures were acclimated to a replete NO_3_^-^ concentration, at a target CO_2_ concentration (380 μatm), saturating light intensity (400 μmol photons m^-2^ s^-1^) and optimal temperature (26 °C).
(TIF)

S1 Table Physiological parameters (± S.E.) of the Chl*a*-specific light-response curves for gross and net photosynthetic O_2_ evolution of *T*. *erythraeum* IMS101 (*n* = 3). Abbreviations; E_0m_^Chl^, the Chl*a* -specific maximum gross O_2_ evolution rate; P_m_^Chl^, the Chl*a* -specific maximum net O_2_ evolution rate; α_g_^Chl^ and α_n_^Chl^ are the Chl*a* -specific initial slopes the light response curve for net and gross photosynthesis; R_d_^Chl^, the Chl*a*-specific dark respiration rate. The *r*^2^ values of all curve fits were > 0.982. Letters in parenthesis indicate significant differences between CO_2_ treatments (One Way ANOVA, Tukey post hoc test; P < .05); where [B] is significantly greater than [A] and [C] is significantly greater than [B] and [A].
(PDF)

S2 Table Physiological parameters (± S.E.) of the fluorescence light-response curves (FLCs) of *T*. *erythraeum* IMS101 (*n* = 3). Abbreviations; ETR_m_^Chl^, the Chl*a*-specific maximum electron transport rate; α_ETR_^Chl^, the Chl*a*-specific initial slope of the electron transport rate light response curve; β_ETR_^Chl^, the Chl*a*-specific light saturated slope of the electron transport rate light response curve. The *r*^2^ values of all curve fits were > 0.977. Letters in parenthesis indicate significant differences between N-source treatments (One Way ANOVA, Tukey post hoc test; P < .05); where [B] is significantly greater than [A] and [C] is significantly greater than [B] and [A].
(PDF)

S1 File Calculation of inorganic carbon speciation. (PDF)

S2 File Calculation of dissolved inorganic N concentration. (PDF)

S3 File Measuring O_2_ production and consumption. (PDF)

S4 File MIMS sample preparation. (PDF)

S5 File Elemental stoichiometry. (PDF)

S6 File Spectrophotometric chlorophyll *a* analysis. (PDF)

S7 File Spectrally corrected *in vivo* light absorption. (PDF)

## References

[1] Moore CM, Mills MM, Langlois R, Milne A, Achterberg EP, La Roche J, et al Relative influence of nitrogen and phosphorus availability on phytoplankton physiology and productivity in the oligotrophic sub-tropical North Atlantic Ocean. Limnology and Oceanography. 2008;53(1):291–305.

[2] Moore JK, Doney SC, Lindsay K, Mahowald N, Michaels AF. Nitrogen fixation amplifies the ocean biogeochemical response to decadal timescale variations in mineral dust deposition. Tellus B. 2006;58(5):560–72.

[3] Vitousek PM, Howarth RW. Nitrogen limitation on land and in the sea: how can it occur? Biogeochemistry. 1991;13(2):87–115.

[4] Dugdale R, Goering J. Uptake of new and regenerated forms of nitrogen in primary productivity. Limnology and Oceanography. 1967:196–206.

[5] Gruber N, Sarmiento JL. Global patterns of marine nitrogen fixation and denitrification. Global Biogeochemical Cycles. 1997;11(2):235–66.

[6] Coles VJ, Hood RR, Pascual M, Capone DG. Modeling the impact of *Trichodesmium* and nitrogen fixation in the Atlantic Ocean. Journal of Geophysical Research. 2004;109:C06007.

[7] Hood RR, Coles VJ, Capone DG. Modeling the distribution of *Trichodesmium* and nitrogen fixation in the Atlantic Ocean. Journal of Geophysical Research. 2004;109(6):L06301.

[8] Campbell L, Carpenter E, Montoya J, Kustka A, Capone D. Picoplankton community structure within and outside a *Trichodesmium* bloom in the southwestern Pacific Ocean. Vie et milieu. 2005;55(3–4):185–95.

[9] Capone DG, Zehr JP, Paerl HW, Bergman B, Carpenter EJ. *Trichodesmium*, a globally significant marine cyanobacterium. Science. 1997;276(5316):1221–9. doi: 10.1126/science.276.5316.1221

[10] Carpenter EJ, Capone DG. Nitrogen fixation in *Trichodesmium* blooms. Marine Pelagic Cyanobacteria: *Trichodesmium* and other Diazotrophs. 1992;362:211–7.

[11] Zehr JP, Bench SR, Carter BJ, Hewson I, Niazi F, Shi T, et al Globally distributed uncultivated oceanic N_2_-fixing cyanobacteria lack oxygenic photosystem II. Science. 2008;322(5904):1110–2. doi: 10.1126/science.1165340 19008448 10.1126/science.1165340

[12] Zehr JP, Waterbury JB, Turner PJ, Montoya JP, Omoregie E, Steward GF, et al Unicellular cyanobacteria fix N_2_ in the subtropical North Pacific Ocean. Nature. 2001;412(6847):635–7. doi: 10.1038/35088063 11493920 10.1038/35088063

[13] Goering JJ, Dugdale RC, Menzel DW. Estimates of in situ rates of nitrogen uptake by *Trichodesmium* sp. in the tropical Atlantic Ocean. Limnology and Oceanography. 1966:614–20.

[14] Carpenter EJ, McCarthy JJ. Nitrogen fixation and uptake of combined nitrogenous nutrients by *Oscillatoria* (*Trichodesmium*) *thiebautii* in the western Sargasso Sea. Limnology and Oceanography. 1975;20(3):389–401.

[15] Glibert P, Banahan S. Uptake of combined nitrogen sources by *Trichodesmium* and pelagic microplankton in the Caribbean Sea: comparative uptake capacity and nutritional status. EOS. 1988;69(1089):3996–4000.

[16] Mulholland MR, Ohki K, Capone DG. Nitrogen utilization and metabolism relative to patterns of N_2_ fixation in cultures of *Trichodesmium* INIBB1067. Journal of Phycology. 1999;35(5):977–88. doi: 10.1046/j.1529-8817.1999.3550977.x

[17] Mulholland MR, Ohki K, Capone DG. Nutrient controls on nitrogen uptake and metabolism by natural populations and cultures of *Trichodesmium* (Cyanobacteria). Journal of Phycology. 2001;37(6):1001–9.

[18] Mulholland MR, Capone DG. Nitrogen fixation, uptake and metabolism in natural and cultured populations of *Trichodesmium* spp. Marine Ecology Progress Series. 1999;188:33–49.

[19] Zehr JP, Jenkins BD, Short SM, Steward GF. Nitrogenase gene diversity and microbial community structure: a cross system comparison. Environmental Microbiology. 2003;5(7):539–54. 12823187 10.1046/j.1462-2920.2003.00451.x

[20] Großkopf T, LaRoche J. Direct and indirect costs of dinitrogen fixation in *Crocosphaera watsonii* WH8501 and possible implications for the nitrogen cycle. Frontiers in Microbiology. 2012;3(236):1–6. 22833737 10.3389/fmicb.2012.00236 PMC3401090

[21] Berman-Frank I, Lundgren P, Chen YB, Küpper H, Kolber Z, Bergman B, et al Segregation of nitrogen fixation and oxygenic photosynthesis in the marine cyanobacterium *Trichodesmium*. Science. 2001;294(5546):1534–7. doi: 10.1126/science.1064082 11711677 10.1126/science.1064082

[22] Capone DG, O’Neil JM, Zehr J, Carpenter EJ. Basis for diel variation in nitrogenase activity in the marine planktonic cyanobacterium *Trichodesmium thiebautii*. Applied and Environmental Microbiology. 1990;56(11):3532–6. 16348357 10.1128/aem.56.11.3532-3536.1990 PMC185016

[23] Sandh G, Ran L, Xu L, Sundqvist G, Bulone V, Bergman B. Comparative proteomic profiles of the marine cyanobacterium *Trichodesmium erythraeum* IMS101 under different nitrogen regimes. Proteomics. 2011;11(3):406–19. doi: 10.1002/pmic.201000382 21268270 10.1002/pmic.201000382

[24] Küpper H, Ferimazova N, Setlík I, Berman-Frank I. Traffic lights in *Trichodesmium*. regulation of photosynthesis for nitrogen fixation studied by chlorophyll fluorescence kinetic microscopy. Plant Physiology. 2004;135(4):2120–33. doi: 10.1104/pp.104.045963 15299119 10.1104/pp.104.045963 PMC520784

[25] Bergman B, Sandh G, Lin S, Larsson J, Carpenter EJ. *Trichodesmium*–a widespread marine cyanobacterium with unusual nitrogen fixation properties. FEMS Microbiology Reviews. 2012;37(3):286–302. doi: 10.1111/j.1574-6976.2012.00352.x 22928644 10.1111/j.1574-6976.2012.00352.x PMC3655545

[26] Finzi-Hart JA, Pett-Ridge J, Weber PK, Popa R, Fallon SJ, Gunderson T, et al Fixation and fate of C and N in the cyanobacterium *Trichodesmium* using nanometer-scale secondary ion mass spectrometry. Proceedings of the National Academy of Sciences. 2009;106(15):6345–50. doi: 10.1073/pnas.0810547106 19332780 10.1073/pnas.0810547106 PMC2669351

[27] Ohki K, Zehr JP, Falkowski PG, Fujita Y. Regulation of nitrogen-fixation by different nitrogen sources in the marine non-heterocystous cyanobacterium *Trichodesmium* sp. NIBB1067. Archives of Microbiology. 1991;156(5):335–7.

[28] Helbling EW, VillafañE V, Holm-Hansen O. Effects of ultraviolet radiation on Antarctic marine phytoplankton photosynthesis with particular attention to the influence of mixing: Wiley Online Library; 1994.

[29] Doney SC. Oceanography: Plankton in a warmer world. Nature. 2006;444(7120):695–6. doi: 10.1038/444695a 17151650 10.1038/444695a

[30] Karl D, Michaels A, Bergman B, Capone D, Carpenter E, Letelier R, et al Dinitrogen fixation in the world’s oceans. Biogeochemistry. 2002;57(1):47–98.

[31] Villareal TA, Carpenter EJ. Buoyancy regulation and the potential for vertical migration in the oceanic cyanobacterium *Trichodesmium*. Microbial Ecology. 2003;45(1):1–10. doi: 10.1007/s00248-002-1012-5 12481233 10.1007/s00248-002-1012-5

[32] Davis CS, McGillicuddy DJ. Transatlantic abundance of the N_2_-Fixing colonial cyanobacterium *Trichodesmium*. Science. 2006;312(5779):1517–20. doi: 10.1126/science.1123570 16763148 10.1126/science.1123570

[33] McGillicuddy DJ. Do *Trichodesmium* spp. populations in the North Atlantic export most of the nitrogen they fix? Global Biogeochemical Cycles. 2014;28(2):103–14.

[34] Chen YB, Zehr JP, Mellon M. Growth and nitrogen fixation of the diazotrophic filamentous nonheterocystous cyanobacterium *Trichodesmium* Sp. IMS 101 in defined media: evidence for a circadian rhythm. Journal of Phycology. 1996;32(6):916–23.

[35] McKew BA, Davey P, Finch SJ, Hopkins J, Lefebvre SC, Metodiev MV, et al The trade-off between the light-harvesting and photoprotective functions of fucoxanthin-chlorophyll proteins dominates light acclimation in Emiliania huxleyi (clone CCMP 1516). New Phytologist. 2013. 10.1111/nph.12373 23790241

[36] Radmer RJ, Kok B. Photoreduction of O_2_ primes and replaces CO_2_ assimilation. Plant Physiology. 1976;58(3):336–40. 16659674 10.1104/pp.58.3.336 PMC542242

[37] Platt T, Jassby AD. The relationship between photosynthesis and light for natural assemblages of coastal marine phytoplankton. Journal of Phycology. 1976;12(4):421–30. doi: 10.1111/j.1529-8817.1976.tb02866.x

[38] Kolber ZS, Van Dover C, Niederman R, Falkowski P. Bacterial photosynthesis in surface waters of the open ocean. Nature. 2000;407(6801):177–9. doi: 10.1038/35025044 11001053 10.1038/35025044

[39] Johnsen G, Sakshaug E. Biooptical characteristics of PSII and PSI in 33 species (13 pigment groups) of marine phytoplankton, and the relevance for pulse-amplitude-modulated and fast-repetition-rate fluorometry. Journal of Phycology. 2007;43(6):1236–51.

[40] Kromkamp JC, Forster RM. The use of variable fluorescence measurements in aquatic ecosystems: differences between multiple and single turnover measuring protocols and suggested terminology. European Journal of Phycology. 2003;38(2):103–12.

[41] Woźniak B, Dera J, Ficek D, Majchrowski R, Kaczmarek S, Ostrowska M, et al Modelling the influence of acclimation on the absorption properties of marine phytoplankton. Oceanologia. 1999;(41 (2)):187–210.

[42] Küpper H, Andresen E, Wiegert S, Šimek M, Leitenmaier B, Šetlík I. Reversible coupling of individual phycobiliprotein isoforms during state transitions in the cyanobacterium *Trichodesmium* analysed by single-cell fluorescence kinetic measurements. Biochimica et Biophysica Acta (BBA)-Bioenergetics. 2009;1787(3):155–67. 19186173 10.1016/j.bbabio.2009.01.001

[43] Eichner M, Kranz SA, Rost B. Combined effects of different CO_2_ levels and N sources on the diazotrophic cyanobacterium *Trichodesmium*. Physiologia Plantarum. 2014;152(2):316–30. doi: 10.1111/ppl.12172 24547877 10.1111/ppl.12172 PMC4260171

[44] Holl CM, Montoya JP. Interations between nitrate uptake and nitrogen fixation in continuous cultures of the marine Diazotroph *Trichodesmium* (Cyanobacteria). Journal of Phycology. 2005;41(6):1178–83. doi: 10.1111/j.1529-8817.2005.00146.x

[45] Boatman TG, Lawson T, Geider RJ. A Key Marine Diazotroph in a Changing Ocean: The Interacting Effects of Temperature, CO2 and Light on the Growth of *Trichodesmium erythraeum* IMS101. PLoS ONE. 2017;12(1):e0168796 doi: 10.1371/journal.pone.0168796 28081236 10.1371/journal.pone.0168796 PMC5230749

[46] Mulholland MR, Capone DG. The nitrogen physiology of the marine N_2_-fixing cyanobacteria *Trichodesmium* spp. Trends in Plant Science. 2000;5(4):148–53. doi: 10.1016/s1360-1385(00)01576-4 10740295 10.1016/s1360-1385(00)01576-4

[47] Levitan O, Rosenberg G, Setlik I, Setlikova E, Grigel J, Klepetar J, et al Elevated CO_2_ enhances nitrogen fixation and growth in the marine cyanobacterium *Trichodesmium*. Global Change Biology. 2007;13(2):531–8. doi: 10.1111/j.1365-2486.2006.01314.x

[48] Levitan O, Sudhaus S, LaRoche J, Berman-Frank I. The influence of pCO_2_ and temperature on gene expression of carbon and nitrogen pathways in *Trichodesmium* IMS101. PLoS ONE. 2010;5(12):e15104 doi: 10.1371/journal.pone.0015104 21151907 10.1371/journal.pone.0015104 PMC2997788

[49] Brown CM, MacKinnon JD, Cockshutt AM, Villareal TA, Campbell DA. Flux capacities and acclimation costs in *Trichodesmium* from the Gulf of Mexico. Marine Biology. 2008;154(3):413–22.

[50] Berman-Frank I, Cullen JT, Shaked Y, Sherrell RMF, P.G. Iron availability, cellular iron quotas, and nitrogen fixation in *Trichodesmium*. Limnology and Oceanography. 2001;46(6):1249–60.

[51] Berman-Frank I, Quigg A, Finkel ZV, Irwin AJ, Haramaty L. Nitrogen-fixation strategies and Fe requirements in cyanobacteria. Limnology and Oceanography. 2007;52(5):2260–9.

[52] Milligan AJ, Berman-Frank I, Gerchman Y, Dismukes GC, Falkowski PG. Light-dependent oxygen consumption in nitrogen-fixing cyanobacteria plays a key role in nitrogenase protection. Journal of Phycology. 2007;43(5):845–52.

[53] Baker NR, Harbinson J, Kramer DM. Determining the limitations and regulation of photosynthetic energy transduction in leaves. Plant, Cell and Environment. 2007;30(9):1107–25. doi: 10.1111/j.1365-3040.2007.01680.x 17661750 10.1111/j.1365-3040.2007.01680.x

[54] Mulholland MR, Capone DG. Stoichiometry of nitrogen and carbon utilization in cultured populations of *Trichodesmium* IMS101: implications for growth. Limnology and Oceanography. 2001;46(2):436–43.

[55] Guerrero M, Lara C. Assimilation of inorganic nitrogen. The Cyanobacteria. 1987:163–86.

[56] Luque I, Flores E, Herrero A. Molecular mechanism for the operation of nitrogen control in cyanobacteria. The EMBO journal. 1994;13(12):2862–9. 8026471 10.1002/j.1460-2075.1994.tb06580.x PMC395167

[57] Raven JA, Johnston AM. Mechanisms of inorganic-carbon acquisition in marine phytoplankton and their implications for the use of other resources. Limnology and Oceanography. 1991;36(8):1701–14.

[58] Campbell D, Hurry V, Clarke AK, Gustafsson P, Oquist G. Chlorophyll fluorescence analysis of cyanobacterial photosynthesis and acclimation. Microbiology and Molecular Biology Reviews. 1998;62(3):667–83. 9729605 10.1128/mmbr.62.3.667-683.1998 PMC98930

[59] Edwards G, Walker DA. C3, C4: mechanisms, and cellular and environmental regulation, of photosynthesis: Blackwell Scientific Publications; 1983.

[60] Fryer MJ, Andrews JR, Oxborough K, Blowers DA, Baker NR. Relationship between CO_2_ assimilation, photosynthetic electron transport, and active O_2_ metabolism in leaves of maize in the field during periods of low temperature. Plant Physiology. 1998;116(2):571–80. 9490760 10.1104/pp.116.2.571 PMC35114

[61] Carr H, Björk M. A methodological comparison of photosynthetic oxygen evolution and estimated electron transport rate in tropical *Ulva* (*Chlorophyceae*) species under different light and inorganic carbon conditions. Journal of Phycology. 2003;39(6):1125–31.

[62] Suggett DJ, MacIntyre HL, Kana TM, Geider RJ. Comparing electron transport with gas exchange: parameterising exchange rates between alternative photosynthetic currencies for eukaryotic phytoplankton. Aquatic Microbial Ecology. 2009;56:147–62.

[63] Geel C, Versluis W, Snel JF. Estimation of oxygen evolution by marine phytoplankton from measurement of the efficiency of Photosystem II electron flow. Photosynthesis Research. 1997;51(1):61–70.

[64] Flameling IA, Kromkamp J. Light dependence of quantum yields for PSII charge separation and oxygen evolution in eucaryotic algae. Limnology and Oceanography. 1998;43(2):284–97.

[65] Sundberg B, Campbell D, Palmqvist K. Predicting CO_2_ gain and photosynthetic light acclimation from fluorescence yield and quenching in cyano-lichens. Planta. 1997;201(2):138–45.

[66] Kranz SA, Levitan O, Richter KU, Prášil O, Berman-Frank I, Rost B. Combined effects of CO_2_ and light on the N_2_-fixing cyanobacterium *Trichodesmium* IMS101: physiological responses. Plant Physiology. 2010;154(1):334–45. doi: 10.1104/pp.110.159145 20625004 10.1104/pp.110.159145 PMC2938149

[67] Falkowski PG, Wyman K, Ley AC, Mauzerall DC. Relationship of steady-state photosynthesis to fluorescence in eucaryotic algae. Biochimica et Biophysica Acta (BBA)-Bioenergetics. 1986;849(2):183–92.

[68] Morel A. Available, usable, and stored radiant energy in relation to marine photosynthesis. Deep Sea Research. 1978;25(8):673–88.

[69] Breitbarth E, Wohlers J, Klas J, LaRoche J, Peeken I. Nitrogen fixation and growth rates of *Trichodesmium* IMS-101 as a function of light intensity. Marine Ecology Progress Series. 2008;359:25–36.

[70] Letelier RM, Karl DM. Role of *Trichodesmium* spp. in the productivity of the subtropical North Pacific Ocean. Marine Ecology Progress Series. 1996;133:263–73.

[71] Silsbe GM, Oxborough K, Suggett DJ, Forster RM, Ihnken S, Komárek O, et al Toward autonomous measurements of photosynthetic electron transport rates: An evaluation of active fluorescence‐based measurements of photochemistry. Limnology and Oceanography: Methods. 2015;13(3):138–55.

[72] Suggett DJ, MacIntyre HL, Geider RJ. Evaluation of biophysical and optical determinations of light absorption by photosystem II in phytoplankton. Limnology and Oceanography: Methods. 2004;2:316–32.

