# Supplementary figures and images for: The physiological cost of diazotrophy for Trichodesmium erythraeum IMS101
Source: PLoS One. 2018 Apr 11;13(4):e0195638. doi: 10.1371/journal.pone.0195638 (PMC5895029; doi:10.1371/journal.pone.0195638)

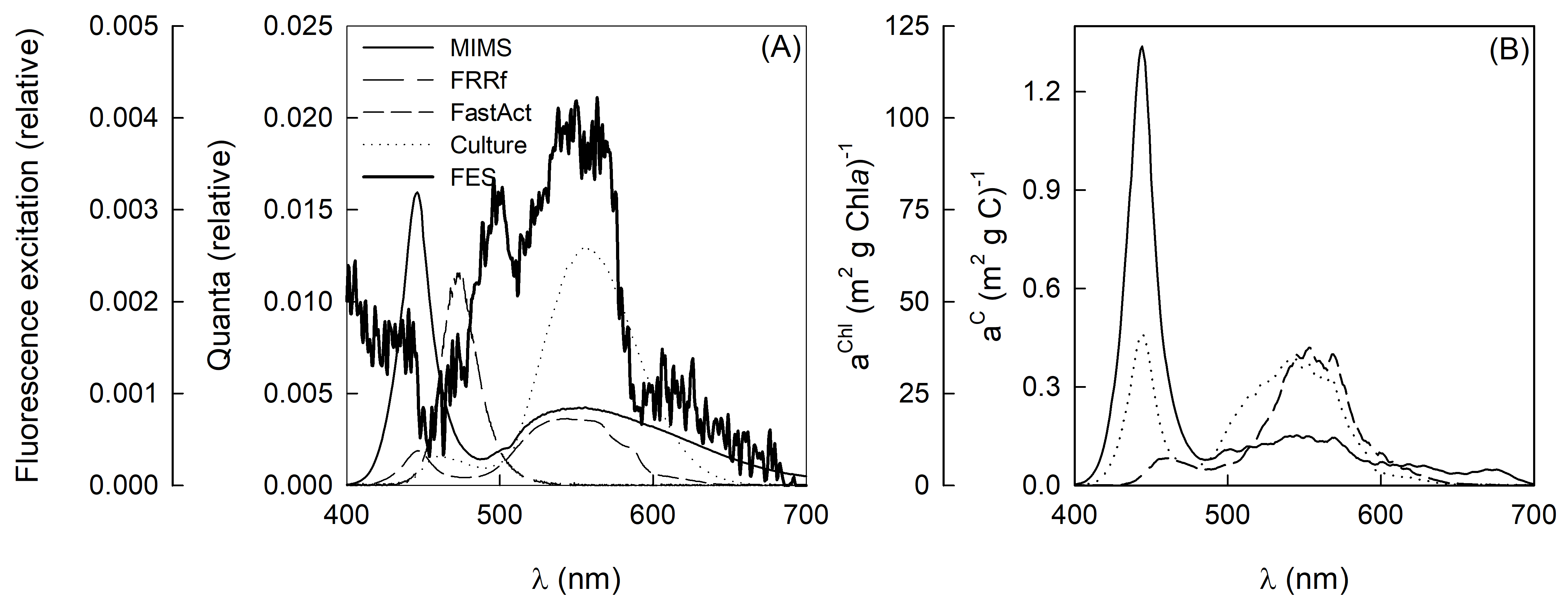

Supplement: S1 Fig — (A) The fluorescence excitation was measured on a 2 mL concentrated sample treated with 20 μM DCMU (final concentration) [71]. Trichodesmium cells were acclimated to 150 μmol photons m-2 s-1 on a 14:10 light:dark cycle, 26 °C and ambient CO2. The sample was measured using a FluorWin fluorometer scanning between 400 to 715 nm at a 1 nm resolution, with the monochromator on the detector set to 730 nm emission [72]. Spectral correction factors were calculated using the FastPro8. (B) An example of an in vivo light absorption spectra of T. erythraeum IMS101 when spectrally corrected to the Culture, MIMS or FRRf LED spectra. (TIF) [file pone.0195638.s001.TIF]

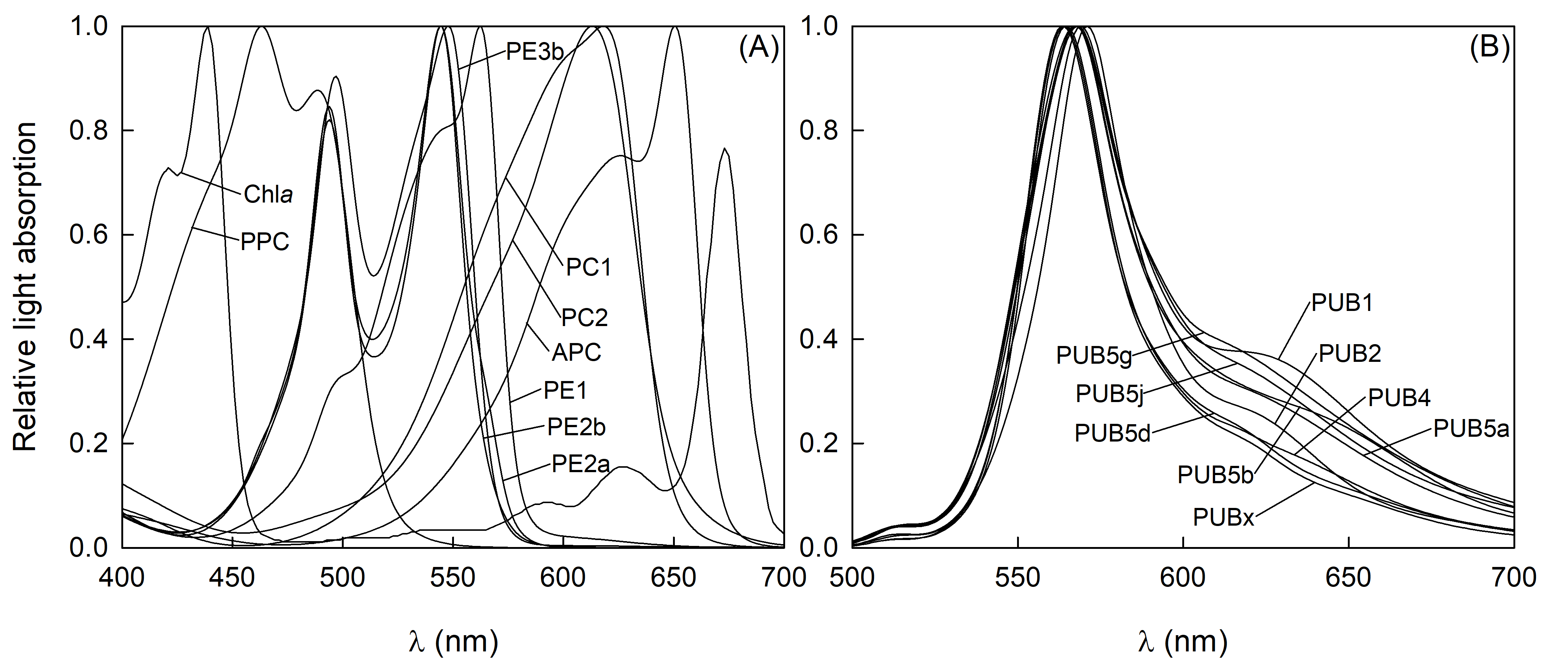

Supplement: S2 Fig — (A) The light absorption spectra of chlorophyll a (Chla), photoprotectant carotenoid (PPC), phycoerythrin (PE), plastocyanin (PC) and alloplastocyanin (APC) pigments. (B) The light absorption spectra of phycourobilin (PUB) pigments. Each pigment spectra was normalised to the maximum peak (λ = 400–700 nm). (TIF) [file pone.0195638.s002.TIF]

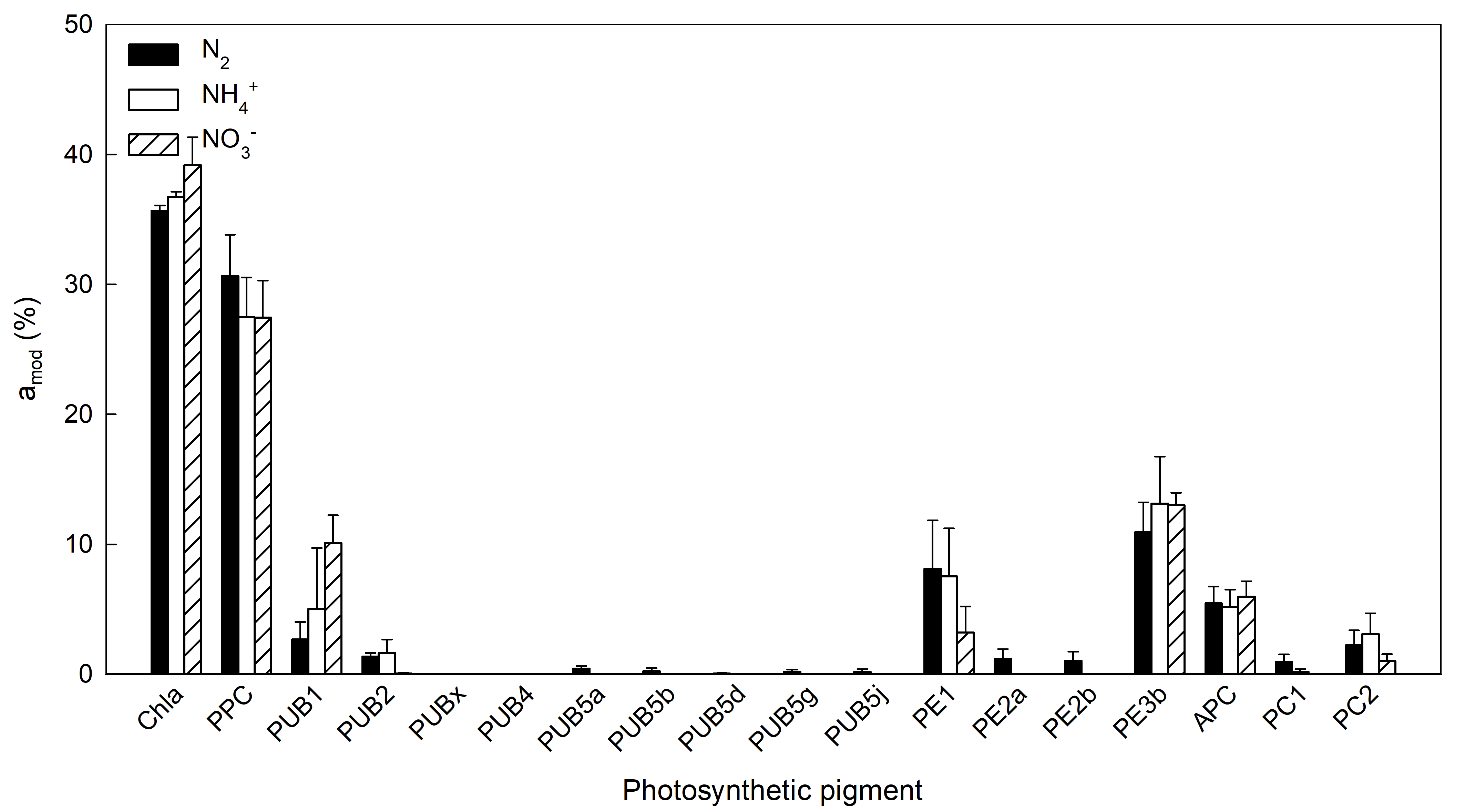

Supplement: S5 Fig — Cultures were acclimated to three N-sources (N2, NH4+ and NO3-), at a target CO2 concentration (380 μatm), saturating light intensity (400 μmol photons m-2 s-1) and optimal temperature (26 °C). Pigments include chlorophyll a (Chla), photoprotectant carotenoid (PPC), phycourobilins (PUB1, PUB2, PUBx, PUB4, PUB5a, PUBb, PUB5d, PUB5g and PUB5j), phycoerythrin (PE1, PE2a, PE2b and PE3b), alloplastocyanin (APC) and plastocyanin (PC1 and PC2). (TIF) [file pone.0195638.s005.TIF]
