# Supplementary material for: The physiological cost of diazotrophy for Trichodesmium erythraeum IMS101
Source: PLoS One. 2018 Apr 11;13(4):e0195638. doi: 10.1371/journal.pone.0195638 (PMC5895029; doi:10.1371/journal.pone.0195638)
Supplement: S1 Table — Abbreviations; E0mChl, the Chla -specific maximum gross O2 evolution rate; PmChl, the Chla -specific maximum net O2 evolution rate; αgChl and αnChl are the Chla -specific initial slopes the light response curve for net and gross photosynthesis; RdChl, the Chla-specific dark respiration rate. The r2 values of all curve fits were > 0.982. Letters in parenthesis indicate significant differences between CO2 treatments (One Way ANOVA, Tukey post hoc test; P < .05); where [B] is significantly greater than [A] and [C] is significantly greater than [B] and [A]. (PDF) [file pone.0195638.s009.pdf]

**Parameters**	**Units**	**N_2**		**NH_4^+**		**NO_3^-**
Gross O_2 evolution						
E_{0m}^{Chl}	mol O_2 (g Chl*a*)$^{-1}$ h^{-1}	0.544 (0.004)	[A]	0.741 (0.065)	[B]	0.594 (0.022)
α_g^{Chl}	mmol O_2 (g Chl*a*)$^{-1}$ h^{-1} (µmol photons m^{-2} s^{-1})$^{-1}$	2.5 (0.5)		3.4 (0.4)		2.7 (0.9)
Net Photosynthesis						
P_m^{Chl}	mol O_2 (g Chl*a*)$^{-1}$ h^{-1}	0.338 (0.021)	[A]	0.579 (0.083)	[B]	0.519 (0.018)
α_n^{Chl}	mmol O_2 (g Chl*a*)$^{-1}$ h^{-1} (µmol photons m^{-2} s^{-1})$^{-1}$	1.5 (0.4)		2.1 (0.3)		2.5 (0.5)
R_d^{Chl}	mol O_2 (g Chl*a*)$^{-1}$ h^{-1}	-0.148 (0.021)		-0.078 (0.015)		-0.063 (0.040)
