# Supplementary material for: The physiological cost of diazotrophy for Trichodesmium erythraeum IMS101
Source: PLoS One. 2018 Apr 11;13(4):e0195638. doi: 10.1371/journal.pone.0195638 (PMC5895029; doi:10.1371/journal.pone.0195638)
Supplement: S2 Table — Abbreviations; ETRmChl, the Chla-specific maximum electron transport rate; αETRChl, the Chla-specific initial slope of the electron transport rate light response curve; βETRChl, the Chla-specific light saturated slope of the electron transport rate light response curve. The r2 values of all curve fits were > 0.977. Letters in parenthesis indicate significant differences between N-source treatments (One Way ANOVA, Tukey post hoc test; P < .05); where [B] is significantly greater than [A] and [C] is significantly greater than [B] and [A]. (PDF) [file pone.0195638.s010.pdf]

**Parameters**	**Units**	**N_2**	**NH_4^+**	**NO_3^-**
ETR_m^{Chl}	mol e^- (g Chl*a*)$^{-1}$ h^{-1}	5.71 (0.59)	4.06 (0.46)	4.69 (0.43)
α_g^{Chl}	mol e^- (g Chl*a*)$^{-1}$ h^{-1} (µmol photons m^{-2} s^{-1})$^{-1}$	0.012 (0.001)	0.010 (0.001)	0.010 (0.001)
β_g^{Chl}	mol e^- (g Chl*a*)$^{-1}$ h^{-1} (µmol photons m^{-2} s^{-1})$^{-1}$	5.76 (0.04) [A]	6.03 (0.01) [B]	5.70 (0.13)
