# Supplementary material for: The physiological cost of diazotrophy for Trichodesmium erythraeum IMS101
Source: PLoS One. 2018 Apr 11;13(4):e0195638. doi: 10.1371/journal.pone.0195638 (PMC5895029; doi:10.1371/journal.pone.0195638)
Supplement: S1 File — (PDF) [file pone.0195638.s011.pdf]

S1 File. Calculation of inorganic carbon speciation.

Prior to the experiment, the carbonate chemistry of all cultures were measured diurnally and the inorganic carbon chemistry defined via *CO2SYS* [1] using: 1st and 2nd equilibrium constants (K1 and K2) for carbonic acid [2], the dissociation constant for KSO_4 [3], the boric acid constant (KB) [4] and total pH. Calculated CO_2 drifts were negligible to the targeted CO_2 concentration (e.g. 380 µatm), verifying that the rate of aeration was sufficient to maintain a CO_2 concentration at high cell densities (S3 Fig). The inorganic carbon chemistry of each replicate culture for all three N-source treatments were measured prior to every dilution with fresh media.

References.

1. Lewis E, Wallace D (1998) CO2SYS Program. Carbon Dioxide Information Analysis Center, Oak Ridge National Laboratory Environmental Sciences Division, Oak Ridge, Tennessee.
2. Millero FJ (2010) Carbonate constants for estuarine waters. Marine and Freshwater Research 61: 139-142.
3. Dickson AG (1990) Thermodynamics of the dissociation of boric acid in synthetic seawater from 273.15 to 318.15 K. Deep Sea Research Part A Oceanographic Research Papers 37: 755-766.
4. Lee K, Kim T-W, Byrne RH, Millero FJ, Feely RA, et al. (2010) The universal ratio of boron to chlorinity for the North Pacific and North Atlantic oceans. Geochimica et Cosmochimica Acta 74: 1801-1811.
