# Supplementary material for: The physiological cost of diazotrophy for Trichodesmium erythraeum IMS101
Source: PLoS One. 2018 Apr 11;13(4):e0195638. doi: 10.1371/journal.pone.0195638 (PMC5895029; doi:10.1371/journal.pone.0195638)
Supplement: S2 File — (PDF) [file pone.0195638.s012.pdf]

S2 File. Calculation of dissolved inorganic N concentrations.

A 2 mL sample of culture was filtered (0.2 µm pore) into 3 mL cryovials and stored in a freezer (-20 C) until analysis. Dissolved inorganic NO_3^- was quantified using the spectrophotometric method as described by Collos et al. [1]; where the absorbance of the filtrate was measured in a quartz cuvette using a Hitachi U-3000 spectrometer between 219 – 220 nm. Dissolved inorganic NH_4^+ was determined using the phenol-hypochlorite method as described by Solorzano [2]; where 1 mL of filtrate was measured spectrophotometrically between 629 – 630 nm.

References.

1. Collos Y, Mornet F, Sciandra A, Waser N, Larson A, et al. (1999) An optical method for the rapid measurement of micromolar concentrations of nitrate in marine phytoplankton cultures. Journal of Applied Phycology 11: 179-184.
2. Solorzano L (1969) Determination of ammonia in natural waters by the phenol hypochlorite method. Limnology and Oceanography 14: 799-801.
