# Supplementary material for: The physiological cost of diazotrophy for Trichodesmium erythraeum IMS101
Source: PLoS One. 2018 Apr 11;13(4):e0195638. doi: 10.1371/journal.pone.0195638 (PMC5895029; doi:10.1371/journal.pone.0195638)
Supplement: S3 File — (PDF) [file pone.0195638.s013.pdf]

S3 File. Measuring O_2 production and consumption.

A quadrupole mass spectrometer (QMG 422, Pfeiffer Vacuum GmbH, Germany) with a cross beam ion source fitted with a tungsten filament was used to ionise the diffused gases. Ions were separated by the mass to charge ratio via electrode rods alternating in polarity, controlled by a high frequency generator (QMH 400-5, Pfeiffer Vacuum GmbH, Germany). The separated ions were collected with a Faraday cup and the signals amplified (EP 422 Pfeiffer Vacuum GmbH, Germany). Multiple detection of the masses 28 (N_2), 32 ($^{16}O_2$) and 36 ($^{18}O_2$) were made repeatedly every 1.2 seconds and the maximum stable reading taken as a discrete measurement. Before and after each set of sample measurements, a series of standard measurements were taken (n = 5) on a flask of air-equilibrated deionised Milli-Q water of known temperature and pressure. The calculation of O_2 evolution and consumption were based on the principle that O_2 was consumed from the media and produced from the photolysis of water. The production of O_2 from the photolysis of water is mainly $^{16}O_2$, as the natural abundance of $^{18}O_2$ is very low (0.2%).
