# Supplementary material for: The physiological cost of diazotrophy for Trichodesmium erythraeum IMS101
Source: PLoS One. 2018 Apr 11;13(4):e0195638. doi: 10.1371/journal.pone.0195638 (PMC5895029; doi:10.1371/journal.pone.0195638)
Supplement: S4 File — (PDF) [file pone.0195638.s014.pdf]

S4 File. MIMS sample preparation.

A 300 mL sample of culture was placed in a large gas tight syringe and gently bubbled with N_2 gas for ~ 20 minutes to lower the $^{16}O_2$ concentration. The headspace was removed and 2 mL of $^{18}O_2$ gas (CK Gas Products, UK - 99% purity) was added and mixed by continuously inverting the syringe for 20 minutes. During this process, the culture was maintained at a low light intensity (< 10 µmol photons m^{-2} s^{-1}) and at growth temperature (26 °C). Samples were incubated using a series of 6 mL glass stopper, gas-tight test tubes, which were cleaned with detergent, acid washed (10% HCl for 1 day) and rinsed with deionised water (Millipore Milli-Q Biocel, ZMQS60FOI) prior to use. Glass beads were placed inside each test tube, allowing the sample to be mixed during incubation (no headspace). The $^{18}O_2$ enriched culture was quickly dispensed into the gas tight glass test tubes, sealed using ground glass stoppers (no headspace) and were immediately placed into a temperature controlled (26 °C) incubator. Light was provided by a white LED light block (Iso Light 400, Technologica, Essex, UK), ranging between 10 to 1100 µmol photons m^{-2} s^{-1}.

A minimum of 10 test tubes were used to determine the initial concentration of O_2 isotopes. An additional 4 test tubes were incubated in the dark (26 °C) to determine dark respiration rates. The initial percentage of $^{18}O_2$ within the cultures were 68%, 60% and 59% for the N_2, NH_4^+ and NO_3^- treatments, respectively. The test tubes were inverted every 10 minutes throughout the incubation to prevent the trichomes settling on the bottom or aggregating at the meniscus.

After incubation, the glass stopper was removed and the sample immediately drawn from the test tube through a stainless-steel coil by a peristaltic pump. The coil consisted of a section of gas permeable membrane enclosed in a gas tight glass sleeve, allowing gases from the sample to diffuse across the membrane and into a glass tube containing a semi-permeable membrane. The glass tube went through a liquid N_2 trap [1] to remove all water vapour prior to entering

the spectrometer. The stainless-steel coil was immersed in a water bath, which maintained the membrane inlet at 26 °C.

References.

1. Kana TM, Darkangelo C, Duane Hunt M, Oldham JB, Bennett GE, et al. (1994) Membrane inlet mass spectrometer for rapid high-precision determination of N_2, O_2, and Ar in environmental water samples. Analytical Chemistry 66: 4166-4166.
