# Supplementary material for: The physiological cost of diazotrophy for Trichodesmium erythraeum IMS101
Source: PLoS One. 2018 Apr 11;13(4):e0195638. doi: 10.1371/journal.pone.0195638 (PMC5895029; doi:10.1371/journal.pone.0195638)
Supplement: S5 File — (PDF) [file pone.0195638.s015.pdf]

S5 File. Elemental stoichiometry.

Three 100 mL aliquots from each culture were vacuum-filtered onto pre-combusted 25 mm (0.45 µm pore) glass fibre filters for measurements of particulate organic carbon (POC), particulate nitrogen (PN) and particulate phosphorus (PP). POC and PN filters were placed in 1.8 mL cryovials (lids off) and dried at 60 °C. POP filters were rinsed with 2 mL of sodium sulphate (0.1 M), placed in a glass 20 mL scintillation vial, 2 mL of magnesium sulphate (0.017 M) added and dried at 60 °C. POC was quantified using a TC analyser (Shimadzu TOC-V Analyser & SSM-5000A Solid Sample Combustion Unit), PN by the method of Bronk and Ward [1] and PP by the method of Solorzano and Sharp [2].

References.

1. Bronk DA, Ward BB (2000) Magnitude of dissolved organic nitrogen release relative to gross nitrogen uptake in marine systems. Limnology and Oceanography 45: 1879-1883.
2. Solorzano L, Sharp JH (1980) Determination of total dissolved phosphorus and particulate phosphorus in natural waters. Limnology and Oceanography 25: 754-758.
