# Supplementary material for: The physiological cost of diazotrophy for Trichodesmium erythraeum IMS101
Source: PLoS One. 2018 Apr 11;13(4):e0195638. doi: 10.1371/journal.pone.0195638 (PMC5895029; doi:10.1371/journal.pone.0195638)
Supplement: S6 File — (PDF) [file pone.0195638.s016.pdf]

S6 File. Spectrophotometric chlorophyll *a* analysis.

Samples were taken for the spectrophotometric determination of chlorophyll *a*, which were then used to normalise light-dependent O_2 evolution rates, PSII electron transport rates and acetylene reduction rates to a Chl*a* basis, as well as to calculate elemental stoichiometry (i.e. Chl*a*:C and Chl*a*:N). A 100 mL sample of culture was vacuum-filtered onto a 25 mm (0.45 µm pore) glass fibre filter (Fisherbrand FB59451, UK) and placed in 5 mL of 100% methanol. Filters were homogenised and extracted overnight at -20 °C, before being centrifuged at 10,000 rpm for 10 minutes and a 3 mL aliquot of the supernatant added to a quartz cuvette. Absorption spectra (400 to 800 nm) was measured using a (Hitachi U-3000, Japan) spectrophotometer and the Chl*a* concentration (µg L^{-1}) calculated using the following equation [1];

$$Chl\ a = \left[\frac{\left(12.9447 \cdot \left(Abs^{665} - Abs^{750}\right)\right) \cdot V_{(E)}}{V_{(F)}}\right] \cdot 1000 \qquad \text{(S1)}$$

where Abs^{665} and Abs^{750} is the baseline-corrected optical density of the methanol extracted sample at 665 and 750 nm; $V_{(E)}$ is the volume of the solvent used for extraction (i.e. 5 mL); $V_{(F)}$ is the volume of culture filtered (i.e. 100 mL); and 12.9447 is a cyanobacteria-specific Chl*a* coefficient for 100% methanol extraction.

References.

1. Ritchie R (2008) Universal chlorophyll equations for estimating chlorophylls a, b, c, and d and total chlorophylls in natural assemblages of photosynthetic organisms using acetone, methanol, or ethanol solvents. Photosynthetica 46: 115-126.
