# Supplementary material for: The physiological cost of diazotrophy for Trichodesmium erythraeum IMS101
Source: PLoS One. 2018 Apr 11;13(4):e0195638. doi: 10.1371/journal.pone.0195638 (PMC5895029; doi:10.1371/journal.pone.0195638)
Supplement: S7 File — (PDF) [file pone.0195638.s017.pdf]

S7 File. Spectrally corrected *in vivo* light absorption.

A 100 mL sample of culture was vacuum-filtered onto a cyclopore (1 µm pore) 47 mm filter (Whatman 60750), re-suspended in 10 mL YBCII media and 3 mL pipetted into a quartz cuvette (1 cm pathlength). Triplicate measurements of the spectral light absorption (400 – 800 nm) were measured using a Hitachi U-3000 spectrophotometer fitted with an integrating sphere (Hitachi High-Technologies, UK). Light absorption spectra were corrected for residual scattering using a linear regression in the 750 – 800 nm region [1] and was normalised to chlorophyll *a* (Chl) and total carbon (C) using the Chl *a*:C ratio of the culture. The light absorption spectra were calculated using the following equation [2];

$$a^{Chl\,(C)}(\lambda) = \frac{OD\,(\lambda)\cdot 2.303\cdot 100}{[Chl\,(C)]} \tag{S2}$$

where $a^{Chl\,(C)}$ (λ) is the Chl*a* (m^2 g Chla^{-1}) or C-specific (m^2 g C^{-1}) light absorption at a specific wavelength (λ = 400 – 700 nm); [Chl*a* (C)] is the cellular chlorophyll *a* or total carbon content (g m^3); OD (λ) is the optical density at a specific wavelength (λ = 400 – 700 nm), 2.303 converts from $\log_{10}$ to the natural log base e and 100 from cm^{-1} to m^{-1}.

The effective light absorption coefficient under the culturing LEDs was determined according to Morel [3];

$$a_{eff}^{Chl\,(C)} = \frac{\sum_{\lambda=400}^{700} E(\lambda)\cdot a^{Chl\,(C)}(\lambda)}{\sum_{\lambda=400}^{700} E(\lambda)} \tag{S3}$$

where $a_{eff}^{Chl\,(C)}$ is the Chl*a* (m^2 g Chla^{-1}) or C-specific (m^2 g C^{-1}) effective light absorption coefficient (λ = 400 – 700 nm); E (λ) is the relative quanta of the culturing LEDs at a specific wavelength (λ = 400 – 700 nm) as determined from the emission spectra (Supporting

Information Fig. S1); $a^{Chl\,(C)}$ is the Chl*a* (m^2 g Chla^{-1}) or C-specific (m^2 g C^{-1}) light absorption coefficient (λ = 400 – 700 nm).

References.

1. Suggett DJ, MacIntyre HL, Geider RJ (2004) Evaluation of biophysical and optical determinations of light absorption by photosystem II in phytoplankton. Limnology and Oceanography: Methods 2: 316-332.
2. Subramaniam A, Carpenter EJ, Karentz D, Falkowski PG (1999) Bio-optical properties of the marine diazotrophic cyanobacteria *Trichodesmium* spp. I. Absorption and photosynthetic action spectra. Limnology and Oceanography 44: 608-617.
3. Morel A (1978) Available, usable, and stored radiant energy in relation to marine photosynthesis. Deep Sea Research 25: 673-688.
